# Global, Regional, and National Death, and Disability-Adjusted Life-Years (DALYs) for Cardiovascular Disease in 2017 and Trends and Risk Analysis From 1990 to 2017 Using the Global Burden of Disease Study and Implications for Prevention

**DOI:** 10.3389/fpubh.2021.559751

**Published:** 2021-10-29

**Authors:** Zhiyong Li, Longfei Lin, Hongwei Wu, Lei Yan, Huanhuan Wang, Hongjun Yang, Hui Li

**Affiliations:** ^1^Institute Chinese Materia Medica China Academy of Chinese Medical Sciences, Beijing, China; ^2^Fengtai District Community Health Center, Beijing, China

**Keywords:** global burden of disease, cardiovascular disease, risk, ischemic heart disease, stroke

## Abstract

**Background:** Cardiovascular disease is the leading cause of death worldwide and a major barrier to sustainable human development. The objective of this study was to evaluate the global, sex, age, region, and country-related cardiovascular disease (CVD) burden, as well as the trends, risk factors, and implications for the prevention of CVD.

**Methods:** Detailed information from 1990 to 2017, including global, regional, and national rates of CVD, and 11 categories of mortality and disability-adjusted life years (DALYs) were collected from the Global Burden of Disease Study 2017. The time-dependent change in the trends of CVD burdens was evaluated by annual percentage change.

**Results:** More than 17 million people died from CVD in 2017, which was approximately two times as many as cancer, and increased nearly 50% compared with 1990. Ischemic heart disease and stroke accounted for 85% of the total age-standardized death rate (ASDR) of CVD. The ASDR and age-standardized DALYs rate (ASYR) of CVD were 1.5 times greater in men compared with women. People over the age of 50 were especially at risk for developing CVD, with the number of cases and deaths in this age group accounting for more than 90% of all age groups. CVD mortality was related to regional economic development and the social demographic index. In regions with a high economic income or socio-demographic index, there was a greater decline in the ASDR of CVD. The ASDR of CVD in high SDI regions decreased more than 50% from 1990 to 2017. Tobacco use, diets low in whole grains, diets high in sodium, and high systolic blood pressure were the important risk factors related to CVD mortality.

**Conclusions:** CVD remains a major cause of death and chronic disability in all regions of the world. Ischemic heart disease and stroke account for the majority of deaths related to CVD. Although the mortality rate for CVD has declined in recent years from a global perspective, the results of CVD data in 2017 suggest that the mortality and DALYs of CVD varied in different ages, sexes, and countries/regions around the world. Therefore, it is necessary to elucidate the specific characteristics of global CVD burden and establish more effective and targeted prevention strategies.

## Introduction

With the rapid development of the world economy and the resulting profound changes in lifestyle, the incidence of chronic diseases is continuing to rise. In particular, the burden of cardiovascular disease (CVD) continues to increase each year (1). CVD is a class of diseases that involve the heart or blood vessels. CVD includes both coronary artery diseases (CAD) such as angina and myocardial infarction, as well as cardiovascular and cerebrovascular diseases such as rheumatic heart disease, ischemic heart disease, stroke, hypertensive heart disease, cardiomyopathy, and myocarditis, among others (2). The age-standardized mortality rate of CVD has been reduced, in recent years, by primary preventive measures (such as lifestyle changes to control risk factors, etc.), widespread use of simple and effective emergency management, and secondary prevention measures. However, despite these effective strategies, CVD remains one of the most serious threats to human life and health and is the first or second leading cause of death in most countries of the world (3, 4).

The Global Burden of Disease (GBD) study was initiated by the World Bank and the WHO in 1991, and before the early 1990s, there was no comprehensive and internally consistent source of information on the global burden of diseases, injuries, and risk factors. The results of GBD were first published in the World Development Report 1993 and the Disease Control Priorities in Developing Countries project (5, 6), then WHO has not only undertaken a major review of the GBD 1990 with its GBD 2000 publications but also provided annual updates in the annex tables of the World Health Report (7). Thus far, the GBD concept has been used by WHO for its report on health information, and in collaboration with external scientists, WHO developed creative new methodologies for the assessment of disease burden resulting from risk factors (8). The GBD approach results in a single summary measure of morbidity, disability, and mortality, the so-called disability-adjusted life year (DALY), which brought new knowledge to the public health community.

The GBD study offers a unique approach within the broader context of global health, for tracking rapidly evolving patterns in CVD epidemiology and their relationship to demographic and socioeconomic changes. One analysis about WHO global health estimates data in 2012 showed that CVD has become the single most important and largest cause of non-communicable disease (NCD) deaths worldwide at over 50% over the last two decades, and it is set to remain the most significant global health burden for decades to come (9). The GBD 2015 study integrated that CVD remained a major cause of premature death and chronic disability for all regions of the world and ischemic heart disease (IHD) and stroke accounted for the majority of health lost to CVD. Sociodemographic changes over the past 25 years have been associated with dramatic declines in CVD in regions with very high sociodemographic index (SDI), but there was only a gradual decrease or no change in most regions (10). The epidemiological report about cardiovascular mortality in global and regional patterns from 1990 to 2013 showed that the number of life years lost to premature CVD deaths is increasing in low- and middle-income regions and the premature death rate due to ischemic heart disease is particularly high in Central Asia. Sub-Saharan Africa and Asia have the largest number of deaths due to stroke (11). Therefore, studying the risk factors and disease burden of CVD has practical significance for the prevention and control of CVD.

## Methods

### Defining Disease Categories

Cardiovascular diseases were classified into 11 types: rheumatic heart disease, ischemic heart disease, stroke, hypertensive heart disease, cardiomyopathy and myocarditis, atrial fibrillation and flutter, aortic aneurysm, peripheral artery disease, endocarditis, non-rheumatic valvular heart disease, and other cardiovascular and circulatory diseases. The full GBD cause hierarchy, including corresponding International Classification of Diseases (ICD)-9 and ICD-10 codes and detailed cause-specific methods, is in GBD 2017 publications on cause-specific mortality 11 and non-fatal health outcomes 12 in the corresponding appendices (4, 12).

### Disability-Adjusted Life Years (DALYs)

Disability-adjusted life years and mortality are two of the major indicators used in the burden of disease studies. DALYs is a measure of overall disease burden, that extends the concept of the potential years of life lost because of premature death to include equivalent years of a “healthy” life lost by being in a state of disability or poor health (13). It was developed in the 1990s as a way of comparing the overall health and life expectancy of different countries and combines the early deaths and disabilities caused by diseases to determine the health of a population. One lost DALY can be thought of as one lost year of a “healthy” life, and the total number of DALY (i.e., the total burden of disease) as a measurement of the gap between the current health of a population and the ideal situation, where everyone in the population lives into old age, in full health (14). DALYs are calculated by taking the sum of years of life lost (YLL) and years lived with disability (YLD), allowing an assessment of the total loss of health from different causes. YLL is calculated by multiplying observed deaths for a specific age in the year of interest by the age specific reference life expectancy estimated using life table methods. YLD is calculated by multiplying disease prevalence (in a number of cases) by a health-state–specific disability weight representing a degree of lost functional capacity. A detailed explanation of the process of disability weight estimation has been reported separately (15, 16).

In this study, we examined the global burden of CVD using the GBD 2017 study by analyzing overall mortality rates and DALYs, as well as the trends in mortality and DALYs by sex and age across 195 countries and territories between 1990 and 2017 (17). Understanding the geographical, temporal, and demographical distribution of the burden of CVD will greatly aid in developing targeted prevention strategies.

### Human Development Index (HDI) and Social Demographic Index (SDI)

The human development index was introduced in 1990 as a new index for measuring development in different communities, used to assess a given country in three key dimensions of human development: a long and healthy life, access to knowledge, and a decent standard of living. HDI is the geometric mean of normalized indices for life expectancy, expected years of schooling (of children), and the gross national income per capita (GNI). The calculated HDI is a number between zero and one. Through this index, the countries around the world are classified into four groups: countries with a very high HDI (HDI ≥ 0.8); countries with a high HDI (0.8 > HDI ≥ 0.7); countries with a medium HDI (−0.7 > HDI ≥ 0.55); and countries with a low HDI (HDI < 0.55) (18). The sociodemographic index (SDI) is a composite indicator of development similar to HDI, and it is estimated to examine changes in CVD burden as a function of the global epidemiological transition. SDI is the equally weighted geometric mean of income per capita, educational attainment, and total fertility rate (1). In this study, the CVD data were collected from 195 countries and territories, which were divided into 5 regions (low, low-medium, medium, high-medium, or high) based on SDI, and were also classified as high, upper middle, lower middle, or low according to HDI.

### Data Source

The annual CVD deaths and DALYs in groups demarcated by sex, territory, country, age, and CVD disease type, as well as the corresponding age-standardized rates, were extracted from the GBD study. In addition, countries were geographically divided into 21 territories, such as East Asia or Central Asia. The use of these various region types to analyze mortality and DALYs is more conducive to the study of regional environment, national ethnicity, living habits, and other factors that may affect CVD incidence and burden. The datasets generated and/or analyzed during the current study are available in the GBD repository (http://ghdx.healthdata.org/gbd-results-tool) (19).

### Statistical Analysis

We used age-standardized death rate (ASDR) and age-standardized DALYs rate (ASYR) to quantify the disease burden of CVD and its types in each region. Standardization is necessary for this study since it eliminates bias in the comparison of ratios or rates. For instance, standardization can eliminate the influences of internal differences (sex, age, etc.) in the subjects of two groups, allowing the analysis of any substantive differences. The age-standardized rates (per 100,000 populations) are calculated directly in this study and are presented with a 95% uncertainty interval (UI). The range of UI values reflects the certainty of an estimate. The 95% UI estimate results of GBD are calculated 1,000 times, each time sampling from distributions rather than point estimates for data inputs, data transformations, and model choice. The 95th uncertainty interval is determined by the 25th and 975th values of the 1,000 values after ordering them from smallest to largest. Larger uncertainty intervals can result from limited data availability, small studies, and conflicting data, while smaller uncertainty intervals can result from extensive data availability, large studies, and data that are consistent across sources. Global CVD mortality from 1990 to 2017 was calculated using the Joinpoint regression model. The National Cancer Institute (NCI) Joinpoint regression program software (version 4.1.0) was utilized to conduct change-trend analyses and identify statistically significant differences, and the annual percentage change (APC) for each trend phase was calculated (20).

## Results

### Global Burden of CVD

**Figure 1:**
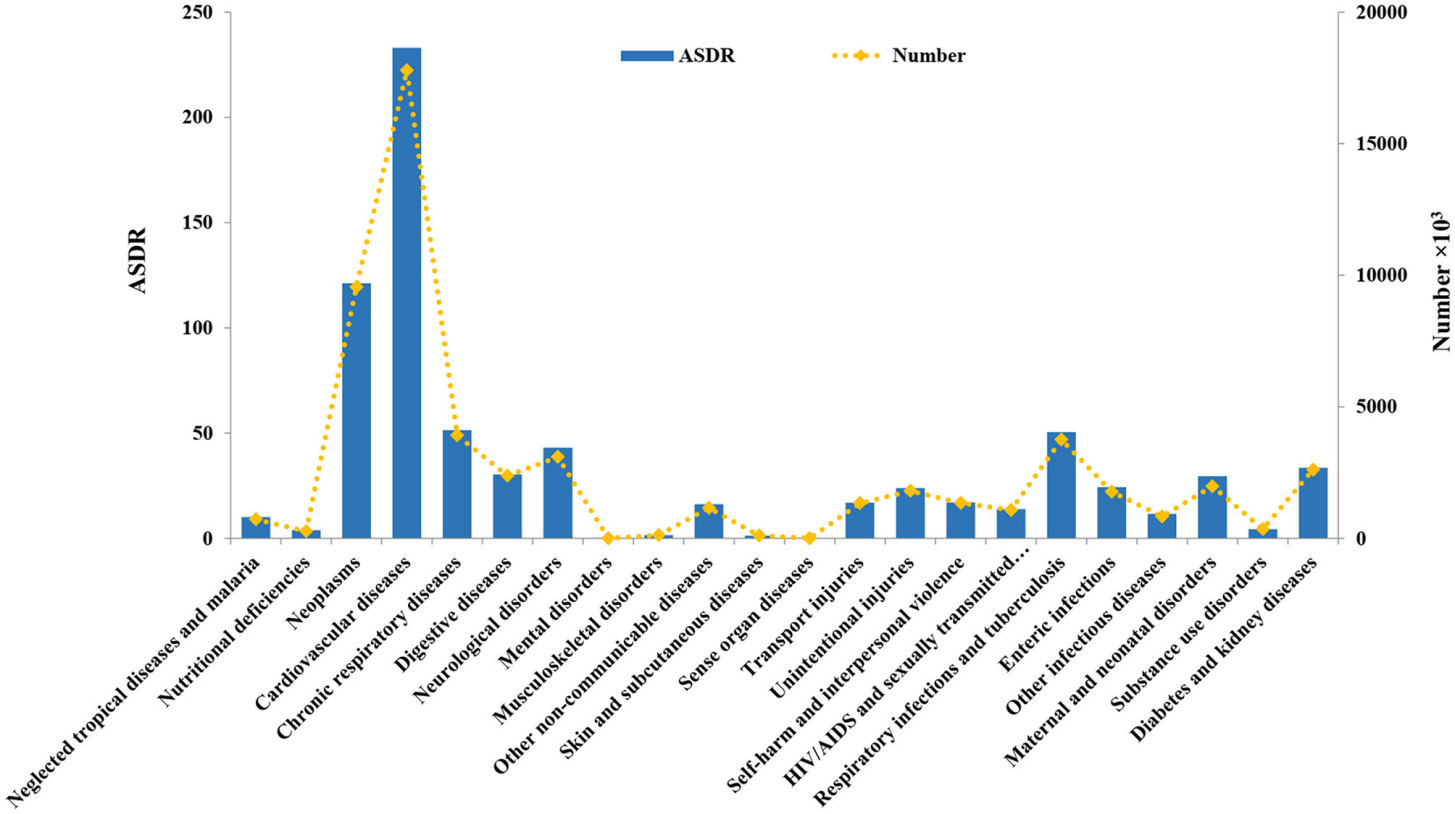
The global age-standardized death rate (ASDR) and death number of 18 types of diseases in 2017.

The diseases included in the GBD study are divided into 18 different types, including neoplasms, cardiovascular diseases, chronic respiratory diseases, digestive diseases, neurological disorders, and mental disorders (4). Among all types of diseases, CVD had the highest ASDR (233.07, and the UI is [229.66–236.38]) and ASYR (4597.93 [4463.74–4734.23], which were both approximately twice as high as the rates for neoplasms (Figure 1). In 2017, more than 17 million (17,790, 949 [17,527,068–18,042,674]) people died from CVD, and there were more than 360 million (365,869,825 [355,162,644–376,747,292]) DALYs attributed to CVD, making CVD the highest burden disease in the world.

The total number of deaths, ASDRs, DALYs, and ASYRs of CVD and its 11 categories from 1990 to 2017 are shown in Table 1. The ASDR and ASYR of CVD in 2017 were significantly decreased compared with 1990 (1990 ASDR: 334.70 [330.50–341.57], 1990 ASYR: 6446.53 [6295.18–6604.55]). However, the total number of deaths and DALYs has increased by nearly 50% since 1990. Ischemic heart disease and stroke were the two largest contributors to disease burden in 2017, with ASDRs of 116.95 [115.13–119.71] and 80.45 [78.86–82.56], respectively, which accounted for 85% of the total ASDR of CVD. The ASYRs of these two types of CVD reached 2132.12 [2093.67–2179.79] and 1657.23 [1587.35–1723.80], respectively. In comparison with 1990, the ASDR and ASYR of most types of CVD were reduced in 2017. The ASDR of rheumatic heart disease had the largest decline, from 8.01 [7.59–8.82] in 1990 to 3.69 [3.43–3.90–3.43] in 2017. The ASDR of peripheral artery disease increased from 0.64 [0.34–1.04] to 0.95 [0.58–1.70] between 1990 and 2017, but the burden of peripheral artery disease was the smallest of all types of CVD, with an ASDR that accounted for only 0.41% of the total CVD ASDR.

**Table 1:** Total number and age-standardized rate of death (ASDR) and disability-adjusted life years (DALYs) of cardiovascular disease (CVD) and its 11 categories in 1990 and 2017.

**Category**	**2017**				**1990**			
	**ASDR**	**Death number × 10^**3**^**	**ASYR**	**DALYs number × 10^**3**^**	**ASDR**	**Death number × 10^**3**^**	**ASYR**	**DALYs number × 10^**3**^**
Cardiovascular diseases	233.07 (236.38–229.66)	17791 (18043–17527)	4597.93 (4734.23–4463.74)	365870 (376747–355163)	334.7 (341.57–330.5)	11942 (12179–11785)	6446.53 (6604.55–6295.18)	266818 (273590–260267)
Rheumatic heart disease	3.68 (3.9–3.43)	286 (303–266)	118.72 (130.66–108.52)	9394 (10333–8577)	8.01 (8.82–7.59)	336 (369–317)	255.43 (278.8–238.84)	12373 (13472–11535)
Ischemic heart disease	116.95 (119.71–115.13)	8930 (9139–8791)	2132.12 (2179.79–2093.67)	170275 (174047–167140)	166.97 (170.76–164.52)	5865 (6000–5772)	2948.56 (3022.05–2889.43)	119480 (122549–116954)
Stroke	80.45 (82.56–78.86)	6167 (6328–6044)	1657.23 (1723.8–1587.35)	132051 (137350–126499)	120.8 (125.26–118.4)	4362 (4520–4272)	2392.71 (2478.87–2316.54)	98876 (102509–95626)
Hypertensive heart disease	12.28 (13.2–8.98)	926 (995–681)	209.42 (226.28–160.54)	16543 (17878–12743)	15.22 (16.52–11.94)	540 (586–425)	275.59 (298.19–218.07)	11079 (12013–8796)
Cardiomyopathy and myocarditis	4.8 (5.03–4.46)	369 (387–342)	130.3 (138.8–120.97)	10247 (10920–9505)	6.47 (6.94–5.74)	239 (258–214)	158.36 (174.07–143.71)	7364 (8124–6677)
Atrial fibrillation and flutter	4 (4.24–3.85)	287 (305–276)	77.97 (92.05–66.14)	5976 (7094–5044)	3.94 (4.18–3.72)	113 (121–108)	80 (95.25–67.59)	2871 (3452–2395)
Aortic aneurysm	2.19 (2.28–2.09)	167 (174–160)	38.18 (40–36.21)	3040 (3186–2877)	2.88 (3.03–2.79)	105 (111–101)	51.09 (54.52–49.01)	2089 (2241–1997)
Peripheral artery disease	0.95 (1.7–0.58)	70 (123–43)	18.39 (27.56–12.46)	1432 (2132–970)	0.64 (1.04–0.34)	20 (32–11)	15.64 (22.7–9.98)	587 (852–378)
Endocarditis	1.1 (1.25–0.98)	83 (94–74)	29.05 (31.66–27.1)	2228 (2423–2082)	1.04 (1.25–0.89)	42 (50–37)	31.48 (37.44–27.59)	1601 (1941–1388)
Non-rheumatic valvular heart disease	1.97 (2.05–1.64)	145 (150–122)	32.74 (35.98–29.53)	2529 (2775–2289)	2.03 (2.31–1.9)	68 (77–64)	37.47 (41.92–34.06)	1488 (1677–1351)
Other cardiovascular and circulatory diseases	4.69 (5.1–4.41)	361 (393–338)	153.82 (176.46–134.75)	12153 (13986–10635)	6.69 (7.51–6.28)	251 (282–236)	200.2 (228.13–176.67)	9010 (10335–7950)

*ASDR (age-standardized death rate) means the ratio of the number of deaths (_n_Dx) in a certain age group (x to x + n) to the average number of people (_n_Px) in that age group, and the calculation formula is ASDR = _n_Dx/_n_Px × 100%0. ASYR (age-standardized DALYs rate) is calculated using the GBD's world population standard. DALY (disability-adjusted life-years)*.

### Global CVD Burden Affected by Sex and Age

**Figure 2:**
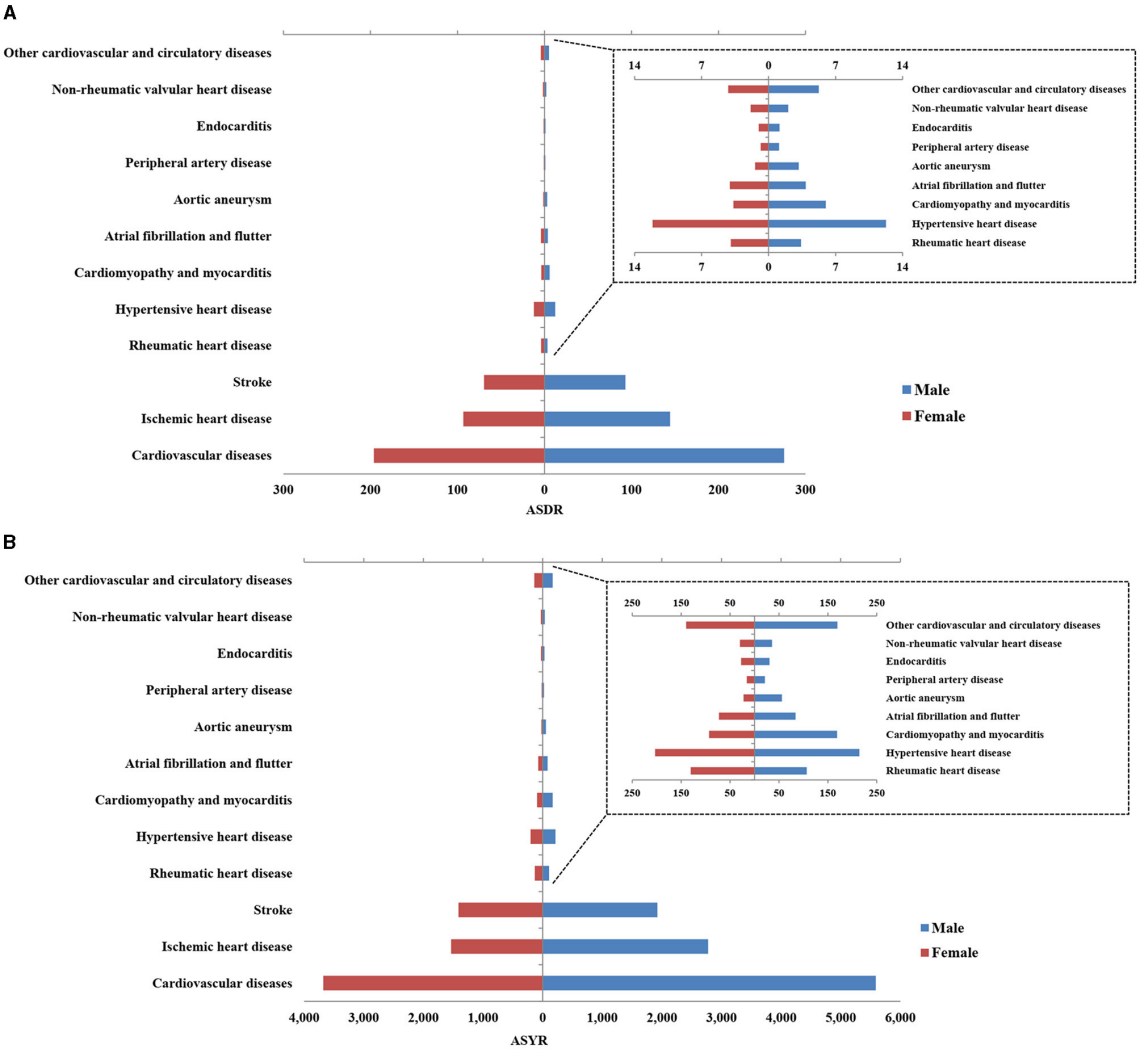
The age standardized of global death **(A)** and disability-adjusted life years (DALYs) **(B)** of cardiovascular disease (CVD) and its 12 categories in 2017 by gender.

The ASDR and ASYR of CVD were 1.5 times higher in men compared with women, as shown in Figure 2 and Supplementary Table 1. In 2017, the total number of global deaths due to CVD was 9,346,335 [9,173,337–9,526,547] in men and 8,444,614 [8,266,536–8,615,992] in women. The ASYRs of men and women were 5588.63 [5428.39–5743.11] and 3680.13 [3541.44–3820.95], respectively. The number of DALYs in men was more than 200 million, compared with more than 150 million in women. Across the 11 types of CVD, aortic aneurysm showed the greatest difference between men and women, with ASDRs and ASYRs in men of 3.15 [2.97–3.35] and 56.00 [52.27–60.17], compared to values in women of 1.41 [1.36–1.52] and 22.34 [21.36–24.74]. Conversely, ASDRs were higher in women compared to men for rheumatic heart disease (3.95 [3.58–4.31] vs. 3.38 [3.19–3.75]) and atrial fibrillation and flutter (4.06 [3.91–4.21] vs. 3.88 [3.46–4.43]). Ischemic heart disease and stroke were the two highest burden diseases in both men and women, with a higher burden in men compared to women. The ASDRs of ischemic heart disease and stroke in men were 144.37 [141.53–147.89] and 92.95 [90.40–95.58], respectively, and in women were 93.32 [91.18–96.05] and 69.60 [67.73–71.89]. The ASYRs of these two diseases were 2776.02 [2715.30–2843.36] and 1924.73 [1848.76–1998.39] in men, and 1534.29 [1487.30–1580.48] and 1412.13 [1336.81–1485.80] in women.

**Figure 3:**
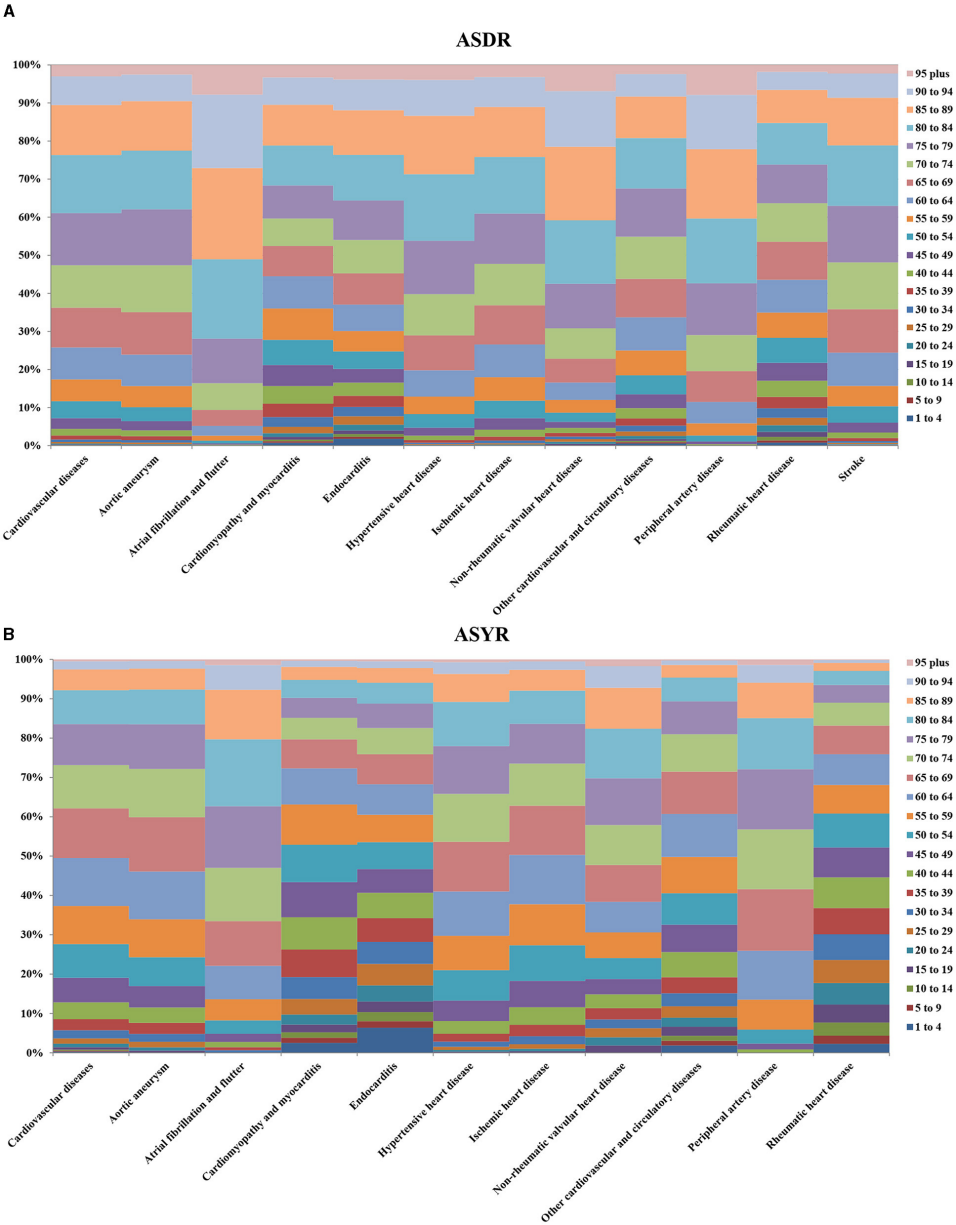
Percentage of global death **(A)** and DALYs **(B)** for CVD and its 11 categories in 2017. The left vertical axis means the percentage of global death and DALYs, and the right vertical axis shows different age groups, the lateral axis represents the different cardiovascular diseases.

CVD data across various age groups in 2017 is presented in Figure 3. People over 50 years old were at the highest risk of developing CVD, and this age group accounted for 93% of all deaths and 81% of all DALYs. There were also differences in the most common types of CVD across the different age groups. Children under 15 years of age mainly suffered from cardiomyopathy and myocarditis, endocarditis, rheumatic heart disease, stroke, and other cardiovascular and circulatory diseases. It is worth noting that the number of CVD deaths and DALYs in children under 1–4 years old was higher than that in children 5–9 years old or 10–14 years old, and the total number of deaths in each group was 11,451 [10,416–12,556], 6671 [6,117–7,283] and 8,959 [8,303–9,636], respectively. Deaths and DALYs from atrial fibrillation and flutter were greatest in individuals 30 years of age and older, and this age group accounted for 99% of all deaths from atrial fibrillation and flutter. Death and DALYs from peripheral artery disease were greatest in individuals 40 years of age and older, and this age group accounted for 98% of all deaths from peripheral artery disease.

### Regional Differences in CVD Burden

The total number of deaths and DALYs, ASDRs, and ASYRs of CVD and its 11 categories in 2017 were tabulated across 21 regions, HDI and SDI regions, as shown in Tables 2, 3 and Supplementary Tables 2, 3. The disease burden varied greatly across different regions. Due to its high population density, Asia had the highest number of CVD deaths and DALYs. In 2017, the total number of deaths due to CVD in East Asia was 4,582,561 [4,439,766–4,723,877] and the number of DALYs was 89,290,189 [85,585,579–93,337,179], which accounted for 25.8 and 24.4% of global CVD deaths and DALYs. Among the 21 regions, CVD burden was the highest in Oceania (ASDR: 516.82 [475.71–562.10], ASYR: 13,011.33 [11,706.73–14,619.79]), Central Asia (ASDR: 535.40 [517.54–554.08], ASYR: 9580.24 [9211.37–9959.80]), and Eastern Europe (ASDR: 452.23 [446.34–458.80], ASYR: 8552.0 [8341.0–8751.0]). The ASDRs of these regions were all approximately 2 times greater than the global level. The CVD burden in the high-income regions of Asia Pacific (ASDR: 79.62 [77.23–81.81]), Australasia (ASDR: 111.06 [103.83–119.17]), Western Europe (ASDR: 120.99 [117.76–124.61]), and Andean Latin America (ASDR: 116.85 [108.75–125.17]) were all relatively light, with ASDR values that were only 50% or less of the global level.

**Table 2:** The age standardized of death of CVD and its 11 categories for different regions in 2017.

**Regions**	**Cardiovascular diseases**	**Rheumatic heart disease**	**Ischemic heart disease**	**Stroke**	**Hypertensive heart disease**	**Cardiomyopathy and myocarditis**	**Atrial fibrillation and flutter**	**Aortic aneurysm**	**Peripheral artery disease**	**Endocarditis**	**Non-rheumatic valvular heart disease**	**Other cardiovascular and circulatory diseases**
East Asia	259.03 (266.68–251.08)	4.01 (4.24–3.83)	105.71 (109.21–102.42)	121.19 (125.31–117.53)	18.38 (20.29–11.71)	1.98 (2.12–1.87)	3.41 (3.55–3.26)	0.92 (1.01–0.83)	0.15 (0.22–0.12)	0.45 (0.47–0.42)	0.7 (0.91–0.66)	2.13 (2.25–1.85)
Southeast Asia	261.47 (271.99–252.62)	1.73 (1.83–1.63)	107.74 (112.62–103.5)	121.71 (126.95–117.03)	13.59 (14.94–10.86)	3.58 (4–3.2)	3.53 (3.77–3.26)	1.83 (2.06–1.64)	0.14 (0.19–0.11)	1.76 (1.94–1.57)	0.68 (0.78–0.62)	5.19 (6.58–4.55)
Oceania	516.82 (562.1–475.71)	24.85 (29.55–20.08)	254.93 (281.68–231.74)	171.56 (191.83–152.08)	26.56 (31.46–18.9)	8.3 (10.3–6.83)	4.51 (4.91–4.09)	4.26 (5.34–3.5)	0.19 (0.24–0.14)	4.92 (6.03–3.88)	2.94 (3.71–2.54)	13.8 (16.92–11.59)
Central Asia	535.4 (554.08–517.54)	4.51 (4.81–4.24)	349.99 (364.05–337.12)	136.64 (141.46–131.78)	19.2 (20.8–14.1)	10.43 (11.37–8.03)	5.13 (5.44–4.8)	2.19 (2.28–2.1)	0.17 (0.27–0.1)	0.52 (0.65–0.47)	0.87 (0.95–0.62)	5.74 (6.04–5.42)
Central Europe	292.69 (298.01–287.68)	1.42 (1.5–1.37)	156.99 (161.81–153.74)	84.86 (87.58–82.77)	15.23 (16.68–10.65)	14.7 (15.4–13.6)	4.47 (4.89–4.3)	2.67 (2.78–2.56)	2.16 (3.89–1.25)	0.65 (0.8–0.58)	2.63 (2.79–1.95)	6.89 (7.53–6.55)
Eastern Europe	452.23 (458.8–446.34)	1.55 (1.63–1.49)	276.86 (283.99–271.97)	124.5 (127.67–122.35)	6.81 (7.39–3.85)	25.58 (26.66–22.92)	4.28 (4.48–4.08)	3.15 (3.26–3.05)	3.34 (5.92–1.92)	0.83 (0.96–0.7)	0.68 (0.72–0.49)	4.64 (4.84–4.45)
High-income Asia Pacific	79.62 (81.81–77.23)	0.96 (1.02–0.92)	32.6 (33.67–31.44)	31.04 (32.03–29.94)	2.61 (4.71–2.29)	1.95 (2.17–1.84)	2.4 (2.7–2.25)	3.8 (3.97–3.61)	0.24 (0.41–0.12)	0.83 (1.26–0.7)	1.73 (1.84–1.22)	1.46 (1.54–1.38)
Australasia	111.06 (119.17–103.83)	1.27 (1.38–1.16)	59.51 (63.97–55.31)	25.74 (27.85–23.79)	2.38 (3.19–1.96)	3.42 (4.32–3.06)	6.58 (7.17–5.82)	3.18 (3.45–2.91)	2.54 (5.13–1.33)	0.97 (1.14–0.76)	3.35 (3.69–2.78)	2.12 (2.32–1.92)
Western Europe	120.99 (124.61–117.76)	1.58 (1.71–1.5)	59.95 (63.65–57.73)	28.98 (30.87–27.95)	6.15 (6.82–3.12)	4.18 (4.4–3.75)	5.48 (6.43–5.2)	2.99 (3.09–2.89)	1.62 (2.87–0.88)	1.42 (1.67–1.09)	3.99 (4.25–3.17)	4.67 (4.9–4.47)
Southern Latin America	171.5 (183.6–160.94)	4.19 (4.6–3.83)	82.83 (89.14–77.11)	44.15 (47.36–41.12)	10.78 (12.8–8.51)	6.27 (7.31–5.69)	4.92 (5.56–4.57)	4.15 (4.53–3.82)	0.63 (1.12–0.36)	2.3 (3.09–1.79)	3.27 (3.74–2.75)	8.02 (8.77–7.26)
High-income North America	146.15 (148.83–143.34)	1.82 (1.89–1.75)	85.87 (88.27–83.5)	28 (28.85–27.08)	6.92 (7.44–4.27)	5.39 (6.17–5.15)	4.75 (5.2–4.38)	2.35 (2.43–2.28)	2.47 (4.92–1.31)	1.61 (2–1.32)	3.37 (3.55–2.75)	3.59 (3.72–3.46)
Caribbean	227.37 (239.71–215.53)	3.41 (4–2.96)	115.19 (121.62–108.98)	68.77 (73.39–64.46)	15.9 (17.7–13.45)	5.29 (5.82–4.64)	4.22 (4.52–3.99)	2.97 (3.25–2.74)	1.77 (3.1–1.08)	1.35 (1.63–1.19)	1.96 (2.19–1.78)	6.53 (7.32–5.95)
Andean Latin America	116.85 (125.17–108.75)	1.52 (1.66–1.39)	60.63 (65.33–56.3)	34.11 (36.68–31.53)	8.12 (9.09–6.68)	1.35 (1.49–1.24)	4.19 (4.51–3.85)	1.48 (1.63–1.34)	0.13 (0.17–0.1)	0.99 (1.12–0.89)	0.7 (0.78–0.64)	3.62 (4.11–3.25)
Central Latin America	152.86 (157.66–148.15)	0.79 (0.84–0.76)	92.6 (96.27–88.34)	35.85 (37.23–34.09)	10.13 (13.99–9.17)	1.85 (1.98–1.69)	4.01 (4.33–3.79)	1.55 (1.64–1.47)	0.63 (1.32–0.34)	0.75 (0.95–0.65)	1.12 (1.21–0.83)	3.56 (3.76–3.38)
Tropical Latin America	178.39 (180.37–176.44)	1.18 (1.23–1.14)	80.44 (82.03–78.47)	56.8 (57.98–55.51)	10.72 (14.16–8.85)	8.43 (9.75–8.01)	4.84 (5.16–4.54)	4.46 (4.63–4.26)	1.72 (3.46–0.86)	1.37 (1.74–1.14)	1.82 (1.91–1.43)	6.6 (6.81–6.39)
North Africa and Middle East	308.37 (318–299.26)	1.59 (2.1–1.35)	193.46 (204.03–185.08)	74.87 (79.62–70.09)	21.7 (24.47–12.77)	2.37 (2.62–2.19)	2.66 (3.1–2.52)	1.51 (1.91–1.38)	0.36 (0.53–0.29)	0.92 (1.01–0.84)	1.63 (1.79–1.49)	7.31 (8.21–5.44)
South Asia	294.56 (305.36–279.06)	9.29 (10.57–8.02)	165.21 (172.39–156.64)	89.48 (93.71–84.53)	12.14 (14.87–9.38)	3.92 (4.95–3)	3.47 (4.12–2.81)	1.91 (2.26–1.58)	0.16 (0.26–0.07)	0.86 (1.1–0.73)	1.32 (1.5–1.11)	6.8 (9.11–5.43)
Central Sub-Saharan Africa	313.81 (344.96–284.05)	5.66 (6.66–4.62)	139.07 (159.5–120.91)	105.71 (120.62–92.09)	36.44 (56.15–20.99)	6.1 (8.93–4.11)	4.79 (6.02–3.67)	2.85 (3.58–2.21)	0.6 (1.23–0.11)	1.87 (2.53–1.21)	1.8 (2.2–1.48)	8.92 (14.7–5.81)
Eastern Sub-Saharan Africa	237.29 (254.3–223.59)	2.71 (3.14–2.27)	99 (111.63–88.3)	88.15 (98.05–78.6)	26.91 (40.58–14.54)	4.37 (5.06–3.18)	3.75 (4.88–2.25)	2.09 (2.67–1.52)	0.42 (0.95–0.08)	1.27 (1.71–0.81)	1.42 (1.8–1.15)	7.2 (11–4.55)
Southern Sub-Saharan Africa	220.15 (229.02–213.03)	3.32 (3.59–3.09)	94.9 (99.25–91.24)	71.65 (75.37–68.69)	24.79 (28.43–22.72)	8.52 (9.1–8.07)	3.33 (3.49–3.04)	2.44 (2.84–2.27)	1.84 (2.45–1.47)	0.89 (0.95–0.83)	1.53 (1.7–1.42)	6.95 (7.48–6.14)
Western Sub-Saharan Africa	230.5 (258.51–207.96)	3.08 (3.51–2.68)	104.04 (117.77–93.22)	86.72 (97.81–78.05)	10.24 (13.56–6.42)	4.2 (5.07–3.38)	4.53 (5.36–3.7)	1.64 (2.02–1.35)	0.38 (0.82–0.12)	1.73 (2.05–1.45)	0.86 (1.07–0.69)	13.08 (18.42–10.17)
High HDI	128.45 (130.71–126.55)	1.52 (1.6–1.47)	67.44 (70.07–66.07)	31.43 (32.72–30.74)	6.02 (6.44–3.82)	4.61 (4.85–4.44)	4.56 (5.18–4.38)	2.98 (3.05–2.9)	1.61 (3.05–0.89)	1.33 (1.64–1.08)	3.22 (3.37–2.54)	3.73 (3.86–3.62)
Upper Middle HDI	254.08 (258.64–249.4)	2.9 (3.04–2.8)	117.76 (120.06–115.68)	101.51 (104.27–99.12)	15.54 (16.84–10.97)	4.94 (5.08–4.63)	3.62 (3.77–3.51)	1.67 (1.76–1.59)	0.71 (1.2–0.47)	0.76 (0.87–0.71)	0.99 (1.09–0.92)	3.66 (3.78–3.44)
Lower Middle HDI	316.88 (325.51–307.01)	6.3 (7.04–5.57)	174.74 (180.28–169.3)	102.59 (106.61–98.77)	13.96 (16.05–11.13)	4.58 (5.25–3.93)	3.7 (4.1–3.25)	1.83 (2.07–1.61)	0.33 (0.46–0.2)	1.02 (1.17–0.91)	1.14 (1.26–1)	6.71 (8.19–5.8)
Low HDI	285.29 (300.16–270.48)	4.83 (5.47–4.4)	128.07 (139.46–118.11)	106.69 (114.76–98.62)	23.77 (33.66–14.82)	4.23 (5.05–3.53)	3.81 (4.57–2.77)	1.94 (2.32–1.54)	0.27 (0.58–0.07)	1.26 (1.65–0.87)	1.35 (1.59–1.18)	9.08 (12.35–6.88)
High SDI	125.7 (127.98–123.74)	1.49 (1.57–1.45)	65.68 (68.09–64.33)	31.3 (32.44–30.62)	5.77 (6.17–3.88)	4.46 (4.68–4.29)	4.51 (5.11–4.33)	2.94 (3.01–2.86)	1.54 (2.9–0.86)	1.28 (1.58–1.03)	3.12 (3.27–2.47)	3.59 (3.72–3.49)
High-middle SDI	292.19 (297.73–286.19)	2.14 (2.24–2.07)	151.4 (154.21–148.65)	103.96 (106.76–101.21)	13.87 (15.02–10.07)	7.49 (7.71–6.91)	3.88 (4.06–3.77)	2.01 (2.11–1.93)	1.19 (2.05–0.75)	0.81 (0.94–0.74)	1.17 (1.28–1.07)	4.27 (4.42–4.07)
Middle SDI	256.81 (262.6–250.58)	3.79 (3.98–3.65)	120.45 (123.25–117.7)	101.78 (105.06–98.54)	16.48 (17.97–11.47)	3.2 (3.39–2.88)	3.59 (3.76–3.41)	1.5 (1.62–1.36)	0.36 (0.56–0.26)	0.9 (0.98–0.83)	0.95 (1.02–0.86)	3.83 (4.14–3.55)
Low-middle SDI	299.71 (311.32–287.76)	6.41 (7.26–5.64)	157.51 (164.39–151.22)	101.16 (106.4–96.54)	15.26 (17.13–11.87)	4.07 (4.59–3.58)	3.67 (4.01–3.27)	1.85 (2.07–1.66)	0.27 (0.38–0.16)	1.01 (1.14–0.91)	1.29 (1.43–1.18)	7.21 (8.65–6.25)
Low SDI	270.56 (282.74–255.78)	8.41 (9.61–7.16)	129.13 (137.45–122.33)	95.89 (101.64–90.89)	17.44 (22.81–12.53)	3.92 (5.11–3.04)	3.31 (4.06–2.6)	1.82 (2.23–1.53)	0.17 (0.36–0.06)	1.05 (1.42–0.82)	1.3 (1.5–1.1)	8.14 (10.71–6.51)

**Table 3:** The age standardized of DALYs of CVD and its 11 categories for different regions in 2017.

**Regions**	**Cardiovascular diseases**	**Rheumatic heart disease**	**Ischemic heart disease**	**Stroke**	**Hypertensive heart disease**	**Cardiomyopathy and myocarditis**	**Atrial fibrillation and flutter**	**Aortic aneurysm**	**Peripheral artery disease**	**Endocarditis**	**Non-rheumatic valvular heart disease**	**Other cardiovascular and circulatory diseases**
East Asia	4558.53 (4762.11–4370.57)	102.9 (116.17–92.22)	1616.55 (1673.39–1561.36)	2332.7 (2457.06–2211.35)	265.54 (292.9–179.41)	59.78 (64.39–55.94)	65.44 (77.92–54.95)	18.06 (19.84–16.27)	8.93 (14.27–5.06)	10.88 (11.65–9.95)	13.17 (16.32–11.98)	64.57 (75.7–54.83)
Southeast Asia	5386.71 (5615.55–5174.46)	72.71 (82.14–65.32)	2114.51 (2210.33–2027.29)	2502.29 (2616.58–2389.66)	261.35 (288.43–207.61)	90.72 (101.37–82.55)	66.17 (78.29–56.54)	31.23 (35.59–28.14)	11.27 (18.42–6.18)	53.4 (58.67–47.68)	16.73 (19.38–14.74)	166.34 (202.48–141.83)
Oceania	13011.33 (14619.79–11706.73)	945.57 (1140.9–762.98)	5997.03 (6839.49–5285.2)	4382.31 (5017.4–3821.03)	611.18 (743.68–406.48)	264.53 (338.54–214.43)	92.47 (105.7–81.23)	91.85 (120.74–70.54)	14.53 (23.22–8.31)	142.53 (183.03–107.41)	76.12 (96.15–61.95)	393.2 (483.83–329)
Central Asia	9580.24 (9959.8–9211.37)	160.99 (177.61–147.04)	5762.45 (6020.98–5516.38)	2664.73 (2789.25–2539.39)	321.61 (353.46–261.99)	309.73 (342.88–230.54)	93.69 (109.66–80.44)	42.18 (44.09–40.42)	8.18 (12.71–4.85)	15.92 (19.33–14.36)	21.23 (24.22–17.03)	179.53 (198.98–162.82)
Central Europe	5013.94 (5199.56–4820.33)	33.11 (34.85–31.67)	2418.91 (2505.27–2353.45)	1556.66 (1635.57–1468.29)	231.07 (256.9–178.94)	283.56 (300.36–256.26)	94.27 (111.89–78.96)	56.05 (58.4–53.43)	36.05 (58.09–23.06)	17.88 (21.38–16.2)	51.64 (56.96–41.83)	234.73 (272.24–201.37)
Eastern Europe	8552 (8751.32–8341)	44.1 (46.45–42.19)	4680.51 (4826.53–4575.58)	2392.55 (2492.75–2281.34)	124.96 (134.79–82.74)	894.26 (937.48–794.65)	90.61 (107.85–76.33)	71.73 (74.43–69.13)	59.78 (98.24–37.39)	32.61 (36.9–28.03)	19.99 (22.65–15.85)	140.91 (151.88–131.39)
High-income Asia Pacific	1577.49 (1687.99–1470.03)	14.02 (14.78–13.42)	530.6 (553.38–507.38)	684.98 (746.02–622.3)	46.08 (70.2–38.37)	61.84 (70.03–55.88)	45.58 (53.54–39.13)	57.55 (60.79–54.23)	6.39 (9.69–3.96)	15.37 (21.9–12.87)	25.25 (29.39–21.35)	89.83 (115.43–69.82)
Australasia	1845.01 (1997.72–1705.3)	24.87 (27.01–22.88)	884.97 (961.16–815.86)	437.39 (475.72–397.12)	36.57 (47.86–31.64)	101.93 (125.5–90.8)	126.25 (151.2–104.5)	48.06 (52.78–43.89)	30.41 (57.17–16.67)	19.24 (22.3–15.74)	44.46 (50–38.93)	90.87 (110.88–75.01)
Western Europe	2044.07 (2162.75–1935.02)	23.58 (25.02–22.53)	915.01 (957.14–878.64)	510.7 (549.13–471.54)	76.49 (84.4–56.26)	89.24 (98–83.29)	101.72 (121.14–85.88)	48.96 (50.87–46.95)	25.66 (40.9–15.79)	24.94 (29.85–20.36)	56.9 (62.48–49.78)	170.87 (207.43–142.36)
Southern Latin America	3192.86 (3443.69–2965.97)	93.94 (106.88–82.74)	1390.84 (1504.64–1282.71)	912.71 (989.94–840.68)	164.34 (199.61–141.98)	146.27 (173.62–132.67)	94 (112.3–78.83)	73.37 (80.34–67.06)	13.16 (20.07–8.53)	45.84 (60.33–38.54)	56.25 (63.8–49.61)	202.15 (230.73–178.3)
High-income North America	2917.77 (3053.56–2791.42)	34.19 (35.74–32.68)	1462.8 (1508.08–1418.81)	670.65 (736.41–604.89)	157.88 (171.51–93.3)	153.63 (175.28–144.73)	119.25 (143.63–98.42)	43.99 (45.64–42.33)	41.15 (74.76–24.3)	34.87 (43.46–30.14)	52.92 (60.05–46.77)	146.44 (170.07–128.16)
Caribbean	4709.93 (5019.75–4431.54)	149.35 (178.71–127.4)	2166.91 (2321.3–2032.3)	1439.75 (1560.29–1341.46)	317.76 (358.66–276.5)	161.41 (182.29–139.62)	88.73 (104.64–75.45)	53.19 (58.26–48.75)	29.5 (45.78–19.33)	44.85 (53.11–39.02)	44.83 (50.54–40.57)	213.66 (243.96–187.01)
Andean Latin America	2361.58 (2542.32–2178.69)	71.71 (87.25–59.26)	1072.99 (1164.61–990.05)	717.63 (775.55–657.96)	143.43 (159.92–118.78)	45.18 (50.28–41.08)	86.36 (102.44–71.81)	27.77 (30.81–24.82)	7.01 (11.16–4.13)	27.2 (31.04–24.26)	18.79 (21.06–16.74)	143.51 (170.9–121.11)
Central Latin America	2930.48 (3063.27–2797.05)	34.8 (40.97–30.12)	1616.27 (1684.51–1548.59)	717.19 (751.21–682.89)	172.17 (226.78–154.18)	59.92 (63.8–56.35)	91.65 (110.44–76.3)	28.56 (30.29–26.87)	15.06 (24.53–9.05)	24.32 (30.98–21.77)	27.3 (29.74–21.57)	143.25 (170–122.97)
Tropical Latin America	3735.26 (3853.05–3620.2)	70.96 (86.91–58.73)	1606.35 (1645.77–1562.08)	1147.94 (1188.17–1111.11)	193.43 (251.6–170.23)	217.64 (245.67–206.91)	103.69 (124.05–86.36)	90.65 (94.36–86.45)	30.85 (54.96–18.15)	40.7 (52.44–35.7)	39.55 (42.28–33.37)	193.51 (214.66–176.57)
North Africa and Middle East	6069.91 (6325.26–5833.91)	70.33 (87.84–58.9)	3620.02 (3813.09–3444.68)	1547.16 (1647.84–1444.3)	363.57 (407.59–228.68)	80.58 (89.26–73.56)	52.17 (61.91–43.95)	29.17 (36.71–26.58)	11.62 (16.91–7.87)	23.33 (25.61–21)	36.96 (41.61–33.22)	235 (272.69–185.26)
South Asia	6006.72 (6222.4–5746.42)	266.77 (302.06–231.44)	3310.22 (3444.72–3169.98)	1786.97 (1869.22–1701.1)	203 (248.2–155.9)	101.1 (127.94–79.06)	65.73 (79.17–54.01)	34.47 (41.02–28.6)	8.36 (13.32–4.77)	21.29 (27.26–18.16)	27.44 (31.29–22.85)	181.38 (232.47–150.47)
Central Sub-Saharan Africa	6081.53 (6709.8–5495.7)	198.24 (231.32–164.7)	2560.13 (2902.75–2243.15)	2073.01 (2343.59–1809.4)	614.58 (898.07–362.57)	176.29 (250.1–123.79)	79.7 (97.47–64.38)	56.49 (72.25–43.77)	16.58 (27.44–7.01)	50.02 (67.68–34.81)	37.62 (44.26–31.69)	218.88 (356.69–150.93)
Eastern Sub-Saharan Africa	4745.39 (5044.04–4465.17)	122.05 (146.01–100.95)	1865.46 (2064.52–1692.66)	1782.83 (1939.45–1619.31)	452.4 (653.14–249.5)	139.47 (161.73–104.82)	66.21 (83.03–47.46)	41.08 (50.83–29.88)	14.09 (22.67–6.38)	37.77 (49.99–25.57)	30.79 (37.31–26)	193.23 (281.68–135.77)
Southern Sub-Saharan Africa	4258.86 (4450.8–4091.96)	149.77 (174.37–130.39)	1713.46 (1792.39–1639.95)	1375.47 (1449.97–1312.75)	415.37 (486.26–384.99)	198.81 (214.17–186.23)	62.42 (73.58–53.21)	46.42 (53.12–42.85)	40.96 (53.23–32.25)	27.93 (30.28–25.82)	33.8 (38.39–31.01)	194.45 (216.88–162.66)
Western Sub-Saharan Africa	4488.48 (5023.71–4061.95)	115.69 (135.63–97.77)	1819.11 (2068.13–1629.24)	1696.91 (1905.04–1540.8)	210.78 (280.47–134.22)	113.36 (136.13–93.9)	70.66 (84.49–58.74)	30.7 (37.66–25.37)	12.77 (20.88–6.85)	59.1 (70.04–49.99)	25.1 (31.18–19.99)	334.28 (459.46–264.29)
High HDI	2414.08 (2534.51–2300.81)	26.65 (27.76–25.9)	1117.83 (1151.36–1091.49)	652.22 (703.88–600.07)	104.37 (113.88–74.45)	116.67 (126.99–110.83)	94.29 (111.95–79.15)	49.96 (51.25–48.55)	26.99 (44.84–17.45)	26.69 (32.16–22.95)	48.55 (53.78–42.66)	149.86 (179.81–125.97)
Upper Middle HDI	4627.74 (4793.92–4475.14)	84.2 (95.62–75.08)	1913.16 (1959.68–1871.44)	1966.65 (2062.56–1873.27)	239.17 (259.26–179.56)	144.95 (149.76–133.75)	72.3 (85.93–60.97)	32.45 (34.23–30.76)	16.79 (24.61–11.5)	21.65 (24.39–20.13)	20.88 (23.13–19.28)	115.53 (131.84–102.22)
Lower Middle HDI	6264.13 (6450–6066.17)	194.63 (216.91–173.2)	3299.78 (3400.65–3200.36)	2046.25 (2122.76–1968.42)	245.98 (281.63–195.6)	119.11 (136.77–103.4)	68.36 (81.07–57.59)	33.74 (38.45–29.9)	11.38 (16.86–7.22)	30.46 (33.57–27.67)	25.72 (29.04–22.4)	188.72 (223.42–162.9)
Low HDI	5725.55 (5984.82–5444.17)	173.68 (196.36–154.15)	2420.78 (2589.44–2261.48)	2153.69 (2280–2005.59)	417.02 (566–262.49)	129.28 (152.99–108.09)	67.36 (81.78–53.2)	38.83 (47.21–31.15)	11.88 (18.83–6.13)	40.44 (53.4–29.09)	31.12 (35.98–27.52)	241.46 (321.54–187.1)
High SDI	2338.64 (2454.53–2226.93)	25.4 (26.36–24.74)	1064.96 (1096.95–1039.89)	650.32 (703.03–597.09)	99.6 (108.78–72.58)	112.87 (122.97–107.31)	93.5 (111.15–78.55)	49.11 (50.43–47.71)	26.08 (43.24–16.87)	25.25 (30.45–21.57)	46.68 (51.89–40.92)	144.88 (174.43–121.29)
High-middle SDI	5254.88 (5432.63–5081.42)	61.23 (68.88–55.61)	2448.99 (2502.54–2393.05)	1998.38 (2103.52–1899.97)	209.7 (227.29–165.2)	220.88 (228.59–201.6)	75.67 (89.74–64.19)	39.64 (41.49–37.83)	23.23 (34.56–15.85)	22.34 (25.32–20.65)	23.5 (26.04–21.63)	131.3 (148.6–117.45)
Middle SDI	4865.9 (5028.7–4708.96)	112.63 (125.04–102.6)	2132.05 (2190.53–2078.55)	1994.43 (2080.66–1906.43)	265.57 (289.86–195.45)	84.89 (90.92–75.67)	70.36 (83.48–59.65)	27.72 (30.06–25.02)	11.98 (17.79–7.63)	25.63 (28.15–23.48)	20.38 (22.52–18.34)	120.26 (138.02–105.02)
Low-middle SDI	6126.01 (6359.29–5899.27)	197.12 (219.93–175.72)	3108.56 (3236.38–2983.76)	2065.21 (2163.7–1972.58)	265.95 (302.7–208.64)	111.9 (125.56–100.16)	68.42 (81.02–58.16)	34.89 (39.3–31.54)	10.46 (15.63–6.42)	31.19 (34.53–28.45)	28.82 (32.62–25.64)	203.48 (238.51–176.07)
Low SDI	5586.41 (5818.83–5324.96)	258.35 (294.51–224.15)	2604.01 (2736.12–2486.96)	1919.72 (2018.61–1829)	305.5 (386.89–216.44)	113.09 (143.97–89.16)	63.09 (76.39–51.51)	34.52 (42.72–29)	9.21 (14.52–5.2)	32.58 (43.05–25.8)	28.99 (34.11–24.62)	217.37 (271.57–178.12)

The burden of specific types of CVD varied across different regions, and different types of CVD also demonstrated different within region burden trends. The ASDRs of most types of CVD were higher in Oceania than the global level, with the ASDR of rheumatic heart disease (24.85 [20.08–29.55]) nearly 7 times higher than the global rate. However, the ASDR of peripheral artery diseases (0.19 [0.14–0.24]) was only 1/5 of the global level. The ASDR of ischemic heart disease in Central Asia (349.99 [337.12–364.05]) was three times higher than the global level and had the highest mortality rate compared to all other regions. However, the mortality rate of peripheral artery disease, endocarditis, and non-rheumatic valvular heart disease in Central Asia was less than half of the global level. Similarly, the ASDRs of most types of CVD were higher in Eastern Europe than the global levels, and cardiomyopathy and myocarditis in particular (25.58 [22.92–26.66]) was more than 5 times greater than the global level. However, the ASDRs of rheumatic heart disease (1.55 [1.49–1.63]), hypertensive heart disease (6.81 [3.85–7.39]), endocarditis (0.83 [0.70-0.96]), and non-rheumatic valvular heart disease (0.68 [0.49-0.72]) in Eastern Europe were far below the global levels. The ASDRs of 10 types of CVD in the high-income Asia Pacific were lower than the global level, but the ASDR for aortic aneurysm was 1.7 times higher than the global level. With the exception of atrial fibrillation and flutter, aortic aneurysm, peripheral artery disease, and non-rheumatic valvular heart disease, the ASDRs of all other types of CVD were lower in Australasia than at the global level. The stroke had the highest mortality rate in Oceania, with an ASDR of 171.56 [152.08–191.83] and ASYR of 4382.31 [3821.03–5017.40]. However, the population base in this area was small, and the total number of CVD deaths was only 10,417 (8,987–12,034). The mortality rate of hypertensive heart disease in Central Sub-Saharan Africa was the highest of all regions, with an ASDR of 36.44 [20.99–56.15] and ASYR of 614.58 [362.57–898.07].

In 2017, the largest number of CVD deaths was in the Upper Middle HDI (7,550,851 [7,410,923–7,691,823]) and Lower Middle HDI areas (6,104,616 [5,922,188–6,271,582]). The ASDRs and ASYRs in the Lower Middle HDI region were the highest, at 316.88 [307.01–325.51] and 6264.13 [6066.17–6450.0], respectively. The lowest ASDR (128.45 [126.55–130.71]) and ASYR (2414.08 [2300.81–2534.51]) values were found in the High HDI areas. The burden of ischemic heart disease in Lower Middle HDI areas was the largest, with an ASDR and ASYR of 174.74 [169.30–180.28] and 3299.78 [3200.36–3400.65], respectively.

**Figure 4:**
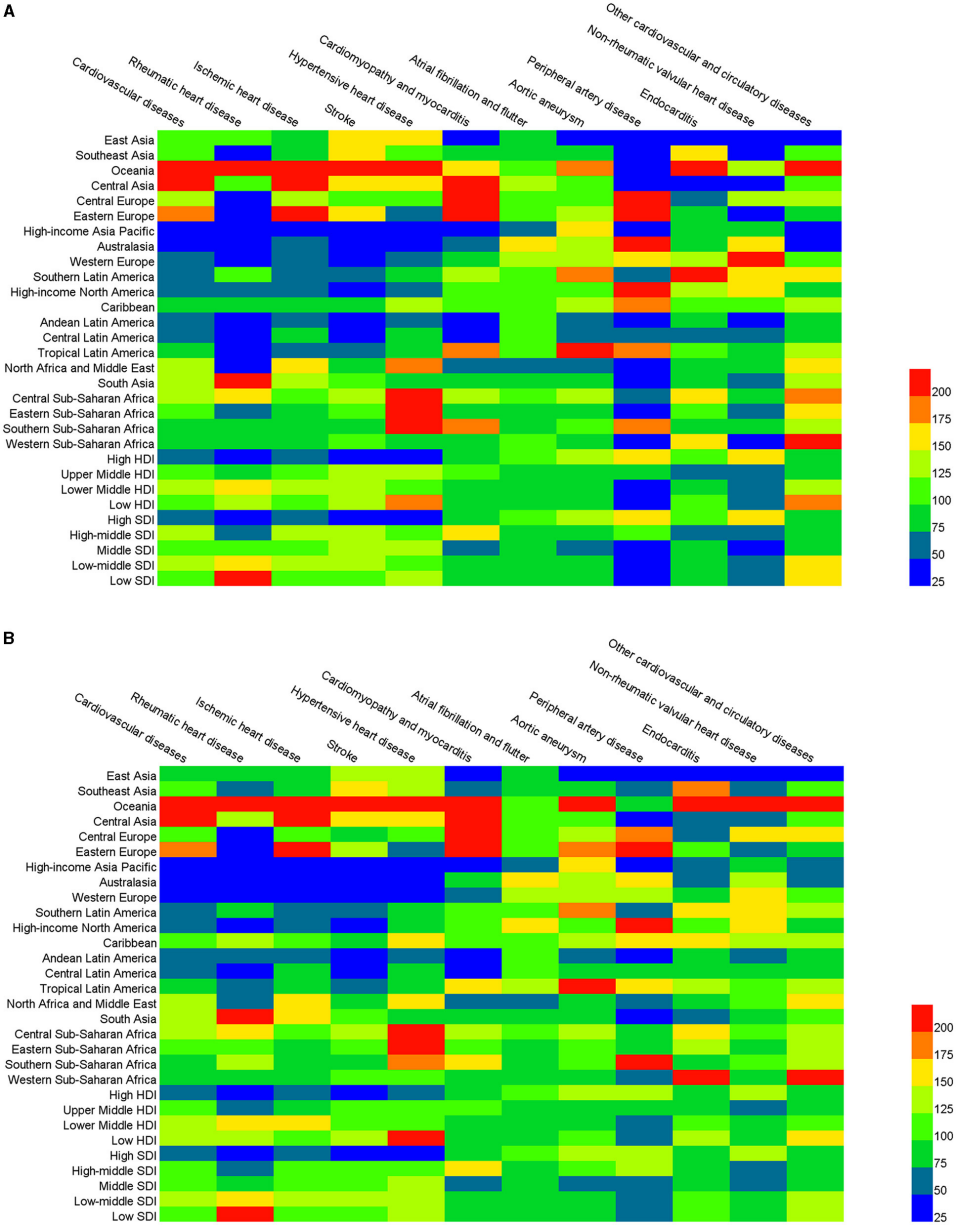
The age-standardized death rate (ASDR) **(A)** and age-standardized DALYs rate (ASRY) **(B)** of CVD and its 11 categories for 21 regions, human development index (HDI), and sociodemographic index (SDI) regions compared with the global in 2017.

The ASDR and ASYR of CVD in High SDI regions were 125.70 [123.74–127.98] and 2338.64 [2226.93–2454.53], only about 50% of the global level. The ASDR and ASYR of CVD in the other four regions were higher than the global level, with the highest rates in the Low-middle SDI region (ASDR: 299.71 [287.76–311.32], ASYR: 6206.01 [5899.27–6359.29]). The ASDRs of rheumatic heart disease (8.41 [7.16–9.61]), hypertensive heart disease (17.44 [12.53–22.81], and other cardiovascular and circulatory diseases (8.14 [6.51–10.71]), were the highest in the Low SDI region. The burden of stroke was lowest in the High SDI region (ASDR: 31.30 [30.62–32.44], ASYR: 650.32 [597.09–703.03]), which were <40% of the global levels. The comparison results are shown in Figure 4.

### Global CVD Burden in Different Countries and Territories

**Figure 5:**
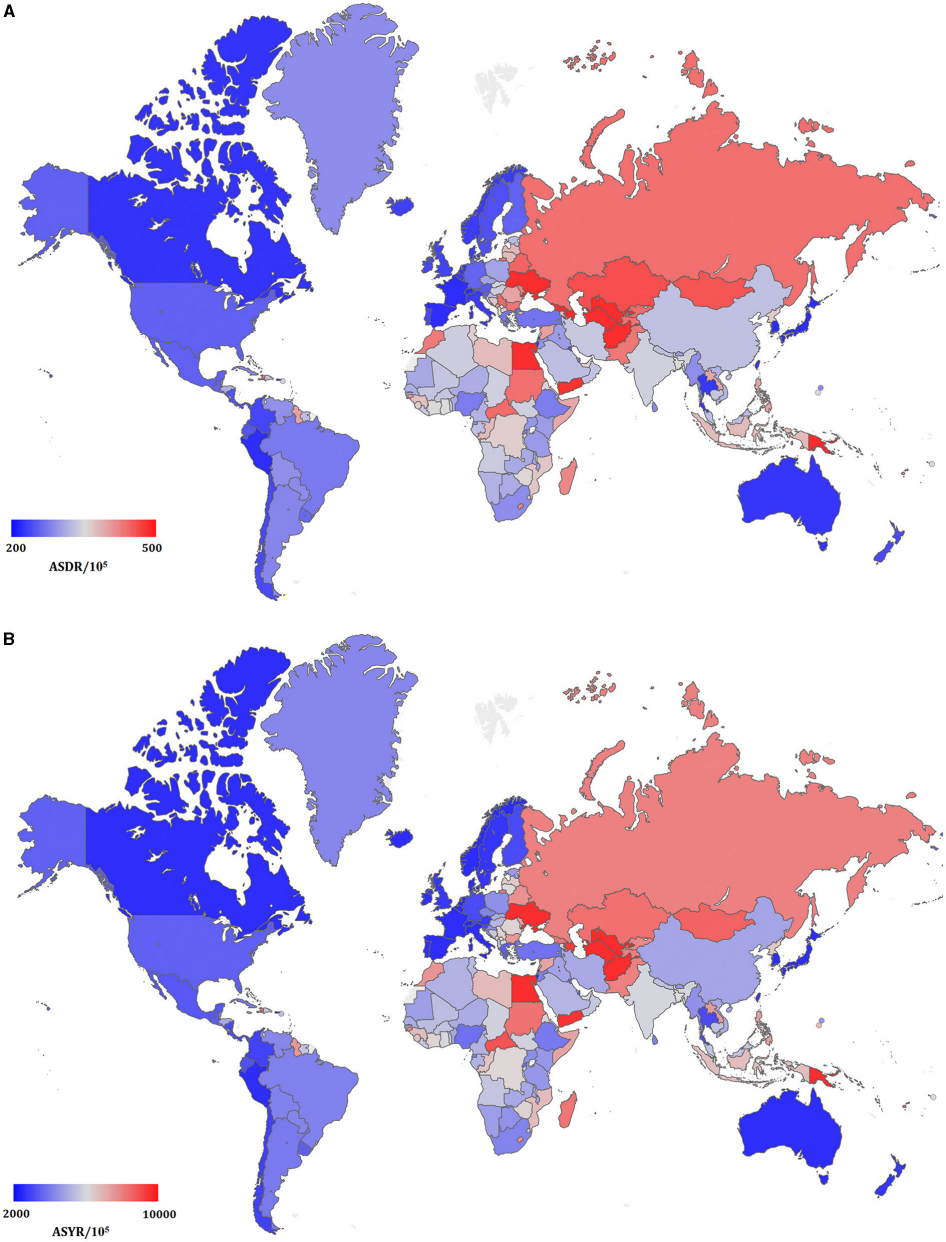
The global disease burden of cardiovascular disease in 195 countries and territories. **(A)** The ASDR of cardiovascular disease in 2017; **(B)** the ASYR of cardiovascular disease in 2017.

The total ASDRs and ASYRs of CVD in 195 countries and territories in 2017 are presented in Figure 5 and Supplementary Table 4. There were 109 countries with CVD ASDRs higher than the global average level across the 195 countries and territories, and the highest of which was Uzbekistan (ASDR: 722.42 [663.68–785.34]). The next highest countries all had ASDRs >500 and included Afghanistan, Papua New Guinea, Azerbaijan, Marshall Islands, Vanuatu, Ukraine, Turkmenistan, and Egypt. The highest CVD ASYR was calculated in Papua New Guinea (14,493.62 [12,714.74–16,598.52]), followed by Afghanistan, Marshall Islands, Vanuatu, Uzbekistan, Turkmenistan, Kiribati, Egypt, Solomon Islands, and Ukraine, all of which also had ASYRs that exceeded 10,000. The countries with the smallest CVD burden as measured by ASDR were Switzerland (99.74 [94.35–105.22]), Spain [99.40 [94.47–104.46]), Israel (93.32 [88.10–98.95]), Singapore (92.24 [87.47–97.58]), France (86.06 [81.81–90.86]), South Korea (86.00 [80.29–91.81]), Peru (85.75 [75.66–96.05]), and Japan (79.37 [77.13–81.43]). The ASYR of Switzerland was the lowest of the above countries, at 1504.54 [1388.53–1616.07].

**Figure 6:**
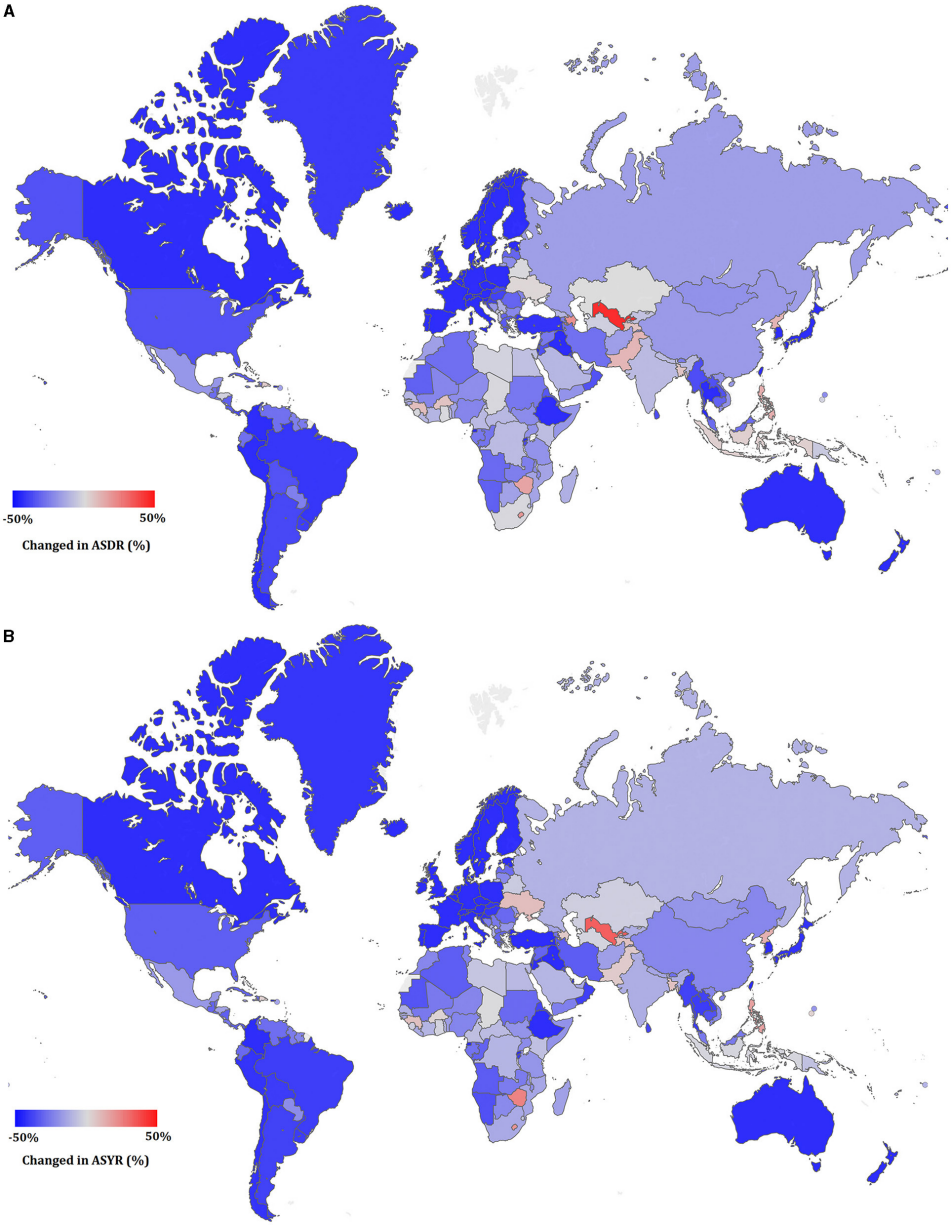
The relative change in ASDR **(A)** and ASYR **(B)** of cardiovascular disease between 1990 and 2017.

The increase in ASDRs and ASYRs between 1990 and 2017 was calculated using the following formula: [(2017ASDR or ASYR) - (1990ASDR or ASYR)]/(1990ASDR or ASYR) × 100 and are shown in Figure 6. Only 16 of the 195 countries/territories showed an increasing trend in CVD ASDR. These countries were Uzbekistan, Azerbaijan, Lesotho, Zimbabwe, Philippines, North Korea, Pakistan, Guinea, Tajikistan, Burkina Faso, Bangladesh, Sao Tome and Principe, Dominican Republic, Indonesia, Ukraine, Gambia, and Samoa. Uzbekistan had the largest increase, with a 59.1% increase in ASDR since 1990. There were 44 countries with a decline in ASDR of more than 50%, of which South Korea decreased the most, to 76.8%. The situation of ASYR was similar to ASDR, with only 15 countries demonstrating an increasing trend since 1990. Uzbekistan again had the largest increase, with a 37.1% increase compared to 1990, and South Korea again had the largest decline (77.4%) since 1990.

**Figure 7:**
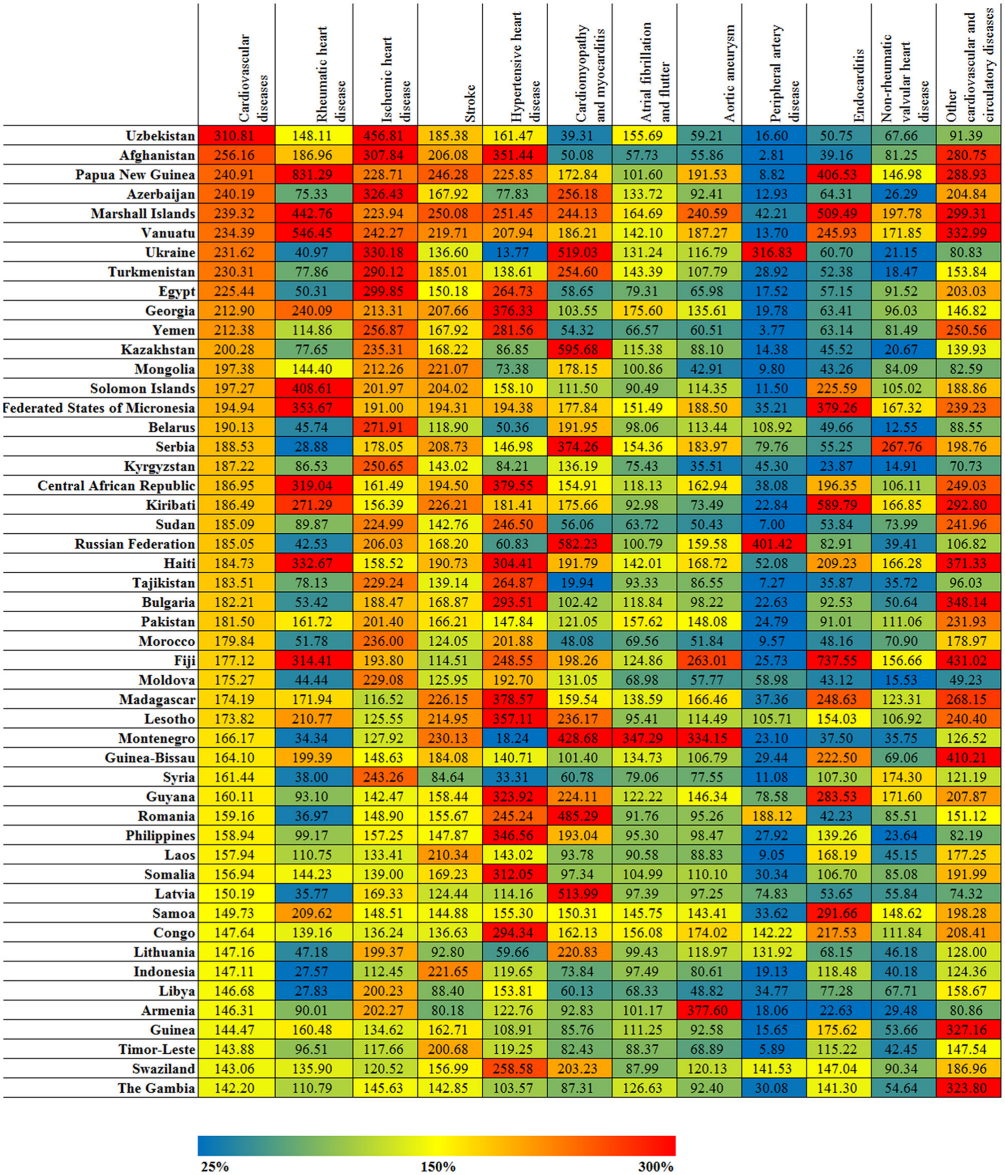
The relative changed (%) in ASDR of cardiovascular disease and its 11 categories for top 50 countries and territories, between 1990 and 2017.

There were varying burdens between different countries and territories for the 11 different types of CVD, as shown in Figure 7. The highest ASDR for ischemic heart disease across all countries and territories was in Uzbekistan (534.24 [488.06–579.99]), which was 4.5 times greater than the average global level. The next highest ASDRs were in Azerbaijan (381.76 [358.70–406.24]) and Ukraine (386.14 [371.42–402.14]), which were 3 times greater than the average global level. The ASDRs of ischemic heart disease were the lowest in Japan (32.97 [31.82–34.07]) and South Korea (34.03 [29.65–34.51]), which were <30% of the average global level. The ASDRs for stroke in Papua New Guinea (198.15 [168.07–228.41]) and Marshall Islands (201.20 [177.56–229.11]) were approximately 2.5 times greater than the average global level. The lowest ASDRs for stroke were found in Singapore (22.03 [20.70–23.70]), Austria (22.65 [21.04–25.14]), France (21.34 [19.93–23.13]), Israel (23.92 [22.32–25.72]), Switzerland (19.51 [17.80–22.34]), and Canada (23.03 [21.65–24.45]), all of which were <30% of the average global level. The ASDR of rheumatic heart disease in Papua New Guinea (30.56 [23.86–37.08]) was more than 8 times greater than the average global level, while in Singapore (0.39 [0.35–0.43]) the ASDR was only 1/10 of the average global level. The ASDR of hypertensive heart disease in Seychelles (59.85 [49.33–72.23]) and Estonia (51.93 [9.76–64.95]) were both 4 times greater than the global average level but did not reach 20% of the average global level in Thailand, Montenegro, Ukraine, Japan, Australia, Andorra, Belgium, Ireland, Israel, Netherlands, Norway, Canada, and Saudi Arabia. The ASDR of cardiomyopathy and myocarditis in Kazakhstan (28.58 [16.50–33.13]) was 6 times greater than the average global level, but their ASDR of non-rheumatic valvular heart disease was only 1/5 of the average global level. The ASDR of aortic aneurysms in Japan was ~2 times the average global level, but the ASDR of the other 10 CVD types was lower than the average global level. The ASYR trends for the 11 CVD types across the different countries and territories were essentially the same as the results for ASDR.

### Trend Analysis

**Figure 8:**
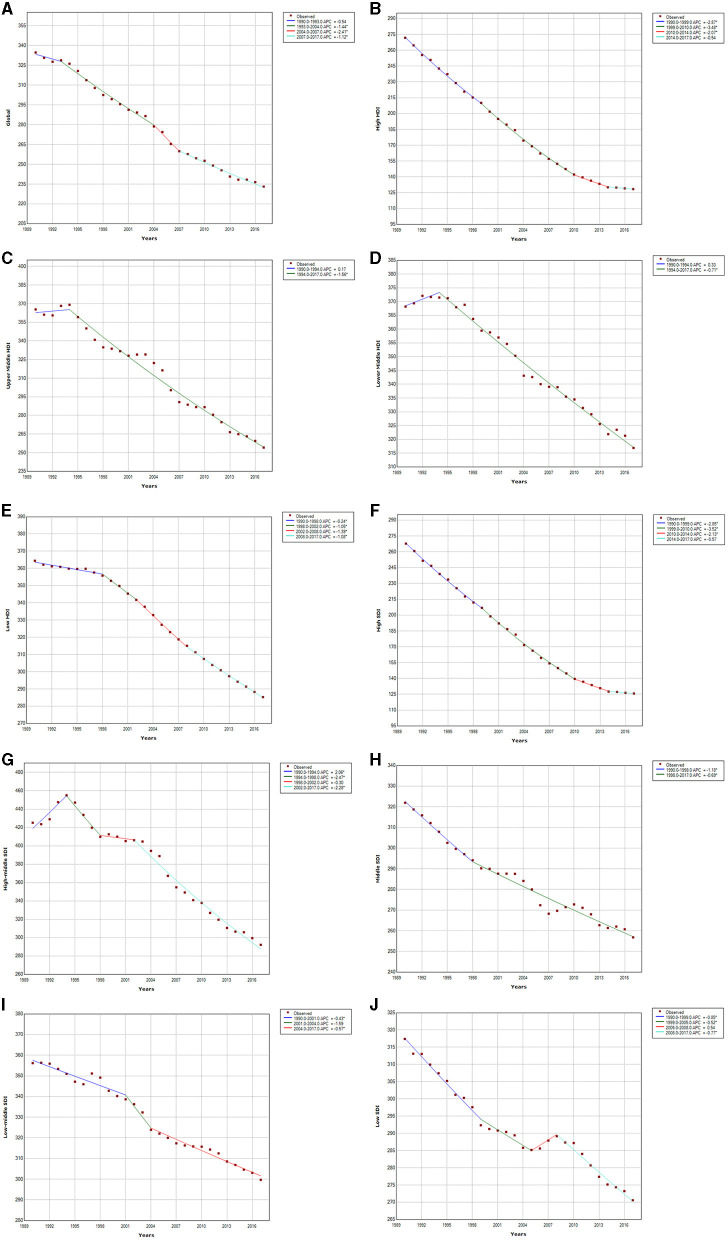
Cardiovascular disease trends in global, HDI, and SDI regions ASDR from 1990 to 2017. Global **(A)**; high HDI **(B)**; upper middle HDI **(C)**; lower middle HDI **(D)**; low HDI **(E)**; high SDI **(F)**; high-middle SDI **(G)**; middle SDI **(H)**; low-middle SDI **(I)**; low SDI **(J)**.

We analyzed the trends in CVD mortality between 1990 and 2017, both globally and in the different economic income and social demographic index regions, as seen in Figure 8. The ASDR of global CVD decreased over the 28 year time period, with the fastest decline rate (APC = −2.41) from 2004 to 2007. The decreasing trend then slowed down and stabilized between 2007 and 2017, and especially from 2013 to 2017, when ASDR decreased by only 3.16%. Trends in changes in the ASDR of CVD also varied between different economic income regions. The High HDI region had the largest decline, with a decrease of 52.7% from 1990 to 2017. The decline rate was the fastest from 1999 to 2010, with an APC of −3.48, and then remained stable from 2014 to 2017. The trends in the Upper Middle and Lower Middle HDI regions were similar. The ASDRs of CVD increased from 1990 to 1994 and then declined until 2017, but the Upper Middle HDI region had a larger decline (30.5% decrease) than the Lower Middle HDI region from 1990 to 2017. The decline in ASDR was slowest in the Lower Middle HDI region (13.9%). The decline in CVD mortality between 1990 and 2017 was positively correlated with the social demographic index. The higher the social demographic index, the greater the decline in CVD mortality. The High SDI region experienced the greatest decline (52.8%) and had a stable trend between 2014 and 2017 (APC = −0.57). There was a rapid increase in ASDR (APC = 2.06) between 1990 and 1994 in the High-middle SDI region, followed by a rapid decrease. There were similar decline trends in the Low middle SDI (14.9% decrease) and Low SDI (13.9%) regions from 1990 to 2017.

### Risk Factor Analysis

**Figure 9:**
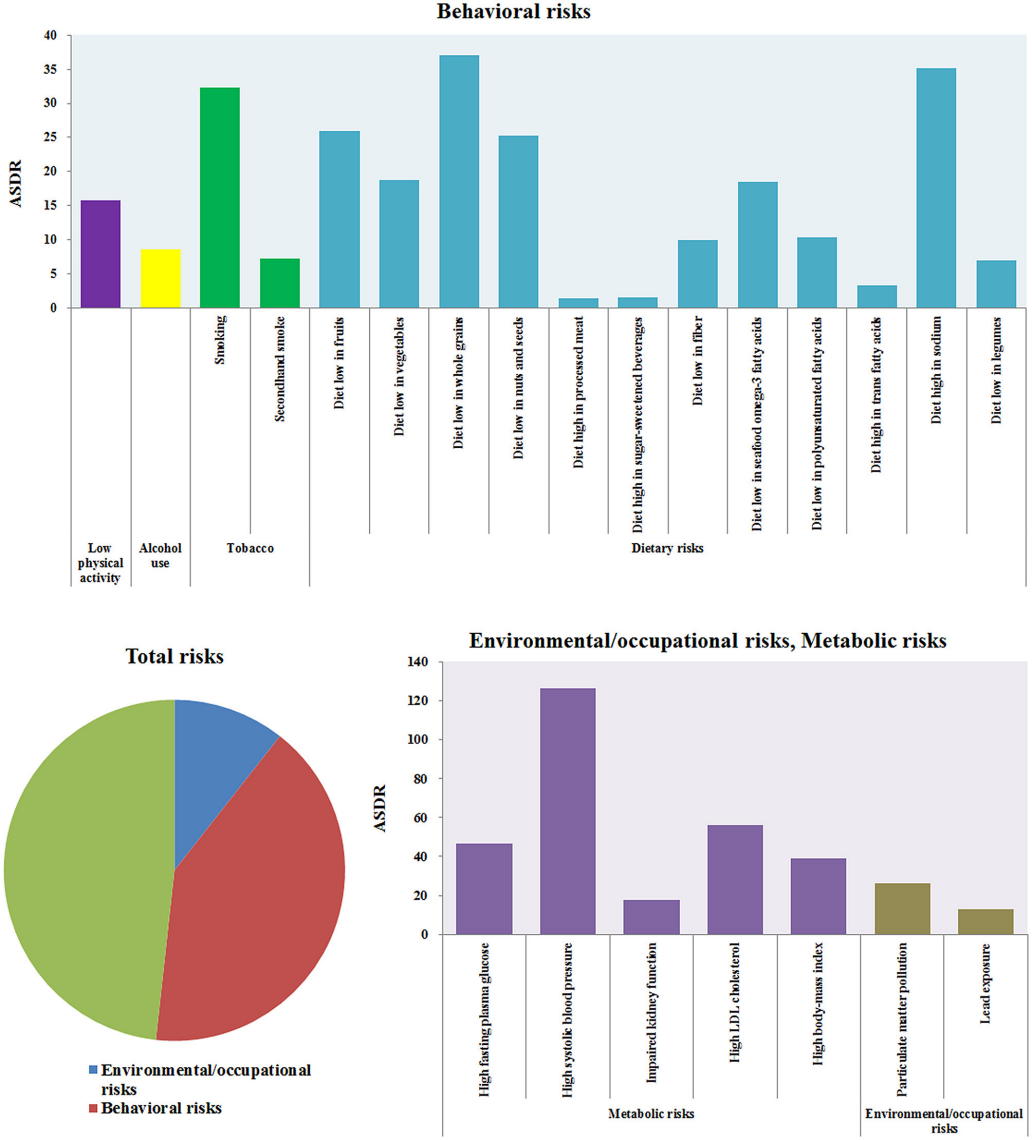
The ASDRs of CVD induced by different risk factors.

The risk factors for CVD could be grouped into three categories: environmental/occupational risks, behavioral risks, and metabolic risks (21, 22). Behavioral risks and metabolic risks were the most important factors for CVD death. The related ASDRs of CVD were 145.27 [137.29–153.09] and 170.45 [158.04–181.73] in 2017, as shown in Figure 9. Environmental/occupational risks mainly consisted of Particulate Matter Pollution (ASDR: 26.21 [23.39–28.98]) and lead exposure (ASDR: 12.91 [8.60–17.60]. Behavioral risks mainly consisted of tobacco use, alcohol use, dietary risks, and low physical activity, of which tobacco (ASDR: 38.36 [36.74–40.23]) and dietary risks (ASDR: 122.63 [112.66–132.27]) were the most influential risk factors. Diets low in whole grains or high in sodium accounted for more than 1/3 of CVD deaths from related dietary risks. Metabolic risks of CVD included high fasting plasma glucose, high systolic blood pressure, impaired kidney function, high LDL cholesterol, Particulate Matter Pollution, and high body mass index. High systolic blood pressure was the greatest of these risk factors (ASDR: 126.22 [112.28–140.37]).

**Figure 10:**
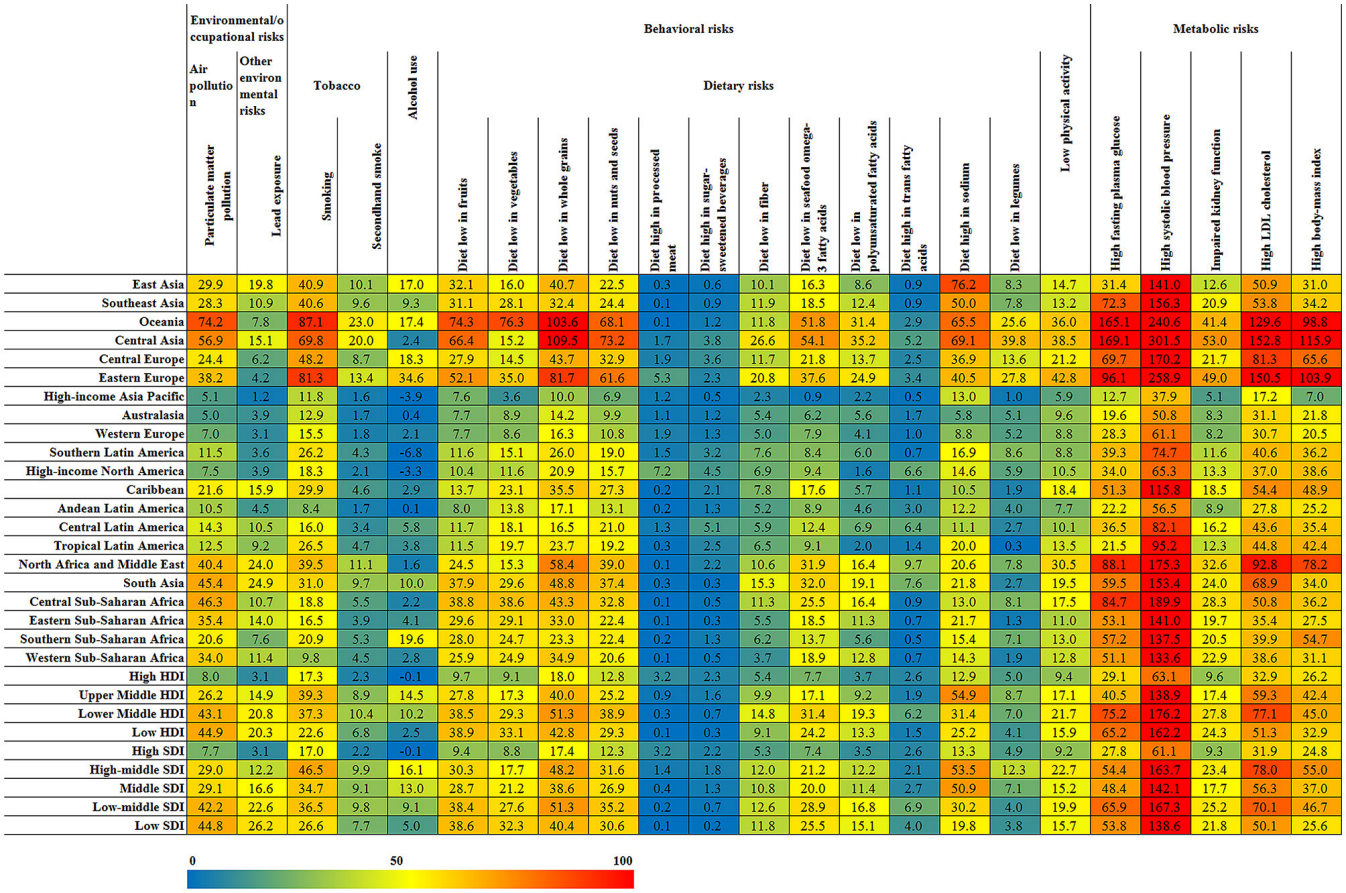
The ASDRs of CVD induced by different risk factors in different regions.

For different regions, Oceania, Central Asia, and Eastern Europe belonged to regions with a higher risk of CVD, while high-income Asia Pacific, Australasia, Western Europe, high-income North America, and Andean Latin America have lower risks, as shown in Figure 10. The standardized mortality rates of CVD caused by Particulate Matter Pollution, smoking, diet low in fruits, diet low in whole grains, High fasting plasma glucose, High systolic blood pressure in Oceania were 74.21 [62.65–86.83], 87.05 [71.40–103.52], 103.63 [72.57–138.67], 165.15 [124.44–218.70] and 240.59 [207.51–276.86], and were 5.12 [4.38–5.86], 11.78 [11.13–12.48], 7.58 [4.39–11.33], 10.00 [6.69–13.77], 12.71 [8.74–18.53], and 37.86 [32.67–43.05] in High-income Asia Pacific, respectively. The death rate of CVD caused by smoking was the highest in Oceania and the lowest in Andean Latin America, and the ASDR was 87.05 [71.40–103.52] and 8.39 [7.28–9.68]. The death rate of CVD caused by alcohol use was the highest, with ASDR at 34.57 [15.57–52.95] in Eastern Europe. The CVD deaths caused by high sodium diets are most common in Asia, the ASDRs in East Asia, Southeast Asia, and Central Asia were 76.22 [44.53–107.53], 49.98 [19.44–83.52], and 69 [13.75–138.43], but was only 5.75 [0.28–16.20] in Australasia. The mortality rate of CVD caused by high systolic blood pressure was the highest in Central Asia (ASDR: 301.46 [259.46–340.99]), followed by Eastern Europe (ASDR: 258.88 [224.17–290.02]). The risk of the high body mass index was also the highest in these two regions, with ASDR at 115.88 [73.24–163.97] and 103.95 [68.34–142.77], respectively. The influence of most risk factors of CVD is negatively correlated with economic income, such as Particulate Matter Pollution, a diet low in fruits, a diet low in vegetables, impaired kidney function, high fasting plasma glucose, high systolic blood pressure, *etc*. However, there also existed another situation in which the mortality caused by Smoking and Diet high in sodium in the Upper Middle HDI region is much higher than the other three regions. Similarly, the risk factors of CVD have the lowest risk in High SDI, and the influence of risk factors and social population index also show regularity and trend.

## Discussion

In this study, we evaluated the burden of CVD in 2017 demarcated by disease type, sex, age, region, and country, and found that the burden of CVD varies widely across these factors. More than 17 million people died from CVD worldwide in 2017, accounting for 30% of the total number of deaths in that year and making CVD the largest burden disease in the world. This conclusion remains highly consistent with previous studies. In 2012, 17.6 million people died of CVD worldwide, this accounted for an estimated 31.43% of global mortality (9). Although the total number of global deaths due to CVD increased by 50% in 2017 compared with 1990, the mortality rate after age standardization has actually decreased by 30% in that time frame, and the ASDR has stabilized in recent years. However, it is worth noting that the changes in CVD mortality are related to regional economic development or social demographic index. In regions with high economic income or high socio-demographic index, the decline in CVD ASDR is greater. The ASDR in the High HDI region decreased by 52.8% from 1990 to 2017 but only decreased by 13.9% in the Lower Middle HDI during that timeframe. Similarly, the ASDR in the High SDI region decreased by 53.1% between 1990 and 2017, while the Low-middle and Low SDI regions only decreased by 15.8 and 14.8%, respectively. Countries such as Uzbekistan, Azerbaijan, Lesotho, Zimbabwe, the Philippines, North Korea, and Pakistan had the greatest CVD burden, which was linked to the lower economic income of those countries. Overall, these results demonstrate the importance of increased investment in the prevention and treatment of CVD for countries in low economic development or social demographic index regions. A study of CVD and associated risk factors also revealed that compared with the mega-countries that hold approximately 50% of the global population, it was necessary to implement well-structured interventions at the primary and secondary care level in the developing countries (23).

The global mortality rate of CVD and most of its types was higher in men than in women, with the exception of rheumatic heart disease and atrial fibrillation and flutter. Previous research has demonstrated that the incidence of coronary heart disease is 10 to 15 years later in women than men. Prior to menopause, the incidence of coronary heart disease in women is only 10 to 30% of that in men. This is due to the protective effect of estrogen on the cardiovascular system in women, which can reduce the incidence of cardiovascular disease (24–26). The risk factors for CVD, including hypertension, hyperlipidemia, diabetes, obesity, and smoking, are also different between women and men, and therefore have a differing influence on CVD incidence and death in women and men (27, 28). For example, low levels of high-density lipoprotein cholesterol (HDL-C) are the risk factors for CVD, and studies have shown that women have a lower risk of CVD than men at any HDL-C level (29, 30). However, the mortality rate of rheumatic heart disease was significantly higher for women than men, suggesting that the prognosis of female rheumatic heart disease is worse than that of male and requires further attention and targeted prevention.

The mortality rate and burden of CVD increased with age. The mortality rate increased after the age of 40, with an increasing rate of change after age 60. Our results indicate that the global CVD burden is highest among individuals older than 50 years of age. It has been previously reported that hypertension, coronary heart disease, hyperlipidemia, hypercoagulability, and cytotoxicity are closely related to aging. Diabetes is also an important risk factor for coronary heart disease (31, 32). The risk of coronary heart disease in diabetic patients is 2–4 times higher than in non-diabetic patients, and the incidence of stroke is approximately 3 times higher (33, 34). With the aging of the global population and the increase in obesity and metabolic syndrome, the elderly should obviously be the major population targeted for prevention efforts. The strict control of blood glucose, blood pressure, and blood lipids are the main preventative measures for reducing the mortality of older patients with CVD (35). In addition, the results of this study showed that children under the age of 15 mainly suffered from cardiomyopathy and myocarditis, endocarditis, other cardiovascular and circulatory diseases, rheumatic heart disease, and stroke. Risk factors for children with CVD were primarily obesity and hypertension (36, 37).

Previous risk factor studies have shown that dietary factors and hypertension are the two largest factors contributing to the burden of CVD disease (38). Among the dietary factors, diets low in whole grains or high in sodium pose the greatest health threat. WHO recommends an adult salt intake of <5 g/day. Smoking is also a major contributor to the burden of CVD. There are approximately 1 billion smokers worldwide, and one in four men and one in 20 women are nicotine dependent (39, 40). The impact of alcohol on cardiovascular disease is still controversial, but it is generally believed that heavy drinking can increase the risk of CVD (41, 42).

Numerous studies have shown that the incidence of CVD in adults and adolescents is high. Therefore, our analysis of the burden of CVD at the sex, age, regional, and national levels help elucidate the characteristics of the global CVD burden. As mentioned in our analysis above, the mortality rate and burden of CVD increased with age, and the global CVD burden is highest among individuals older than 50 years of age, while the age of 15 mainly suffered from cardiomyopathy and myocarditis, endocarditis, or other cardiovascular and circulatory diseases, etc. Smoking is also a major contributor to the burden of CVD. In our study, another important finding is that the changes in CVD mortality are related to regional economic development or SDI. Thus, it is very important and necessary to establish corresponding prevention strategies for CVD. Fortunately, nine necessary strategies to reduce cardiovascular diseases have been proposed in the World Heart Federation's vision (43), and these strategies could cover our conclusions in the mentioned analysis and studies. The following principles should be followed: (1) preventive interventions should begin in childhood and adolescence; (2) establish CVD prevention alliances between non-medical and medical institutions and government agencies in the health field; (3) increase monitoring of mortality, morbidity, and health risks of CVD; (4) strengthen the implementation of tobacco control; (5) implement detection and control of hypertension and secondary prevention of hypertension; (6) strengthen cooperation between different economic incomes and social demographic index regions to establish CVD control; (7) large sample studies in different regions of the world to analyze the characteristics and risk factors of CVD; (8) strengthen the research and development of drugs for the treatment of CVD; (9) improve public health education to promote a reasonable and healthy lifestyle.

## Conclusions

Cardiovascular disease remains a major cause of death and chronic disability in all regions of the world. Ischemic heart disease and stroke account for the majority of the health burden of CVD. Although mortality rates for CVD have declined in recent years from a global perspective, the results of the 2017 CVD data suggest that CVD mortality and DALYs vary widely across different ages, sexes, and countries/regions around the world, particularly in different economic development or social demographic index regions. Additionally, it is necessary to understand and consider the common risk factors that lead to the occurrence of CVD, so that health management and early screening can be encouraged for individuals who are at a high risk of developing CVD. By analyzing the burden of CVD across disease types, sex, age, and regional and national levels, our study helps elucidate the characteristics of the global CVD burden in order to establish more effective and targeted prevention strategies.

### Limitations

Although the GBD estimates can fill in the missing or unavailable data regarding disease burden, some limitations are still worth noting. First, the accuracy of the GBD estimates is mostly dependent on the quality and quantity of the data. These estimates have a high level of uncertainty due to the low proportions of South Americans, Asians, and Africans covered by high-quality CVD registries. Health data on CVD remain extremely limited for some regions of the world, such as sub-Saharan Africa. Second, the GBD study has taken several steps to improve the reliability and comparability of vital registration data, including the redistribution of junk code, but some systematic biases due to the use of regional patterns of diagnostic codes may still exist. Although the inclusion of measurement errors is an important aspect of the GBD study, non-sampling errors have not been quantified. Third, underreporting and misdiagnosis are common during disease registration, especially in developing countries. Fourth, the GBD study accounts for comorbidity using a simulation method that assumes an independent probability of having any disease state. The overall estimate of YLD will increase with age, but CVD is more common in the elderly, so independent assumptions may have a greater impact on this group. Five, the risk factors levels of exposure might be outside the UIs estimated due to less data on some countries or territories, and the estimated results might be inaccurate due to the interaction of multiple risks. Last, the time trends of certain diseases might be affected by changes in diagnostic technology over time.

## Data Availability Statement

The datasets generated and/or analyzed during the current study are available in the GBD repository, http://ghdx.healthdata.org/gbd-results-tool.

## Author Contributions

ZL, LL, HWu, and LY performed the study design, data collection, and wrote the manuscript. ZL revised the manuscript. LL, HWu, and HWa analyzed the data and interpreted the results. HY and HL reviewed and finalized the manuscript and conceptualized the study. All authors have read and approved the final manuscript.

## Funding

This study was financially supported by National Key R&D Plan (2019YFC1708900) and the Fundamental Research Funds for the Central Public Welfare Research Institutes (ZXKT2020023 and ZZ13-YQ-059).

## Conflict of Interest

The authors declare that the research was conducted in the absence of any commercial or financial relationships that could be construed as a potential conflict of interest.

## Publisher's Note

All claims expressed in this article are solely those of the authors and do not necessarily represent those of their affiliated organizations, or those of the publisher, the editors and the reviewers. Any product that may be evaluated in this article, or claim that may be made by its manufacturer, is not guaranteed or endorsed by the publisher.

## Abbreviations

APC: Annual percentage change
ASDR: Age-standardized death rate
ASYR: Age-standardized DALYs rate
CVD: Cardiovascular disease
DALYs: Disability-adjusted of life years
GBD: Global Disease Burden
HDI: Human development index
HDL-C: High-density lipoprotein cholesterol
NCI: National Cancer Institute
TCM: Traditional Chinese Medicine
SDI: Social demographic index
YLD: Years lived with disability
YLL: Years of life lost.
