# Supplementary material for: Global, Regional, and National Death, and Disability-Adjusted Life-Years (DALYs) for Cardiovascular Disease in 2017 and Trends and Risk Analysis From 1990 to 2017 Using the Global Burden of Disease Study and Implications for Prevention
Source: Front Public Health. 2021 Oct 29;9:559751. doi: 10.3389/fpubh.2021.559751 (PMC8589040; doi:10.3389/fpubh.2021.559751)
Supplement: Supplementary file 1 [file Table_1.DOCX]

Table. S1. The Number of global death (A) and DALYs (B) of cardiovascular disease and its 11 categories in 2017 by gender.

| Deaths Number | | | | | | |
| --- | --- | --- | --- | --- | --- | --- |
|  | Male |  |  | Female |  |  |
| Cardiovascular diseases | 9346335 | 9526547 | 9173337 | 8444614 | 8615992 | 8266536 |
| Rheumatic heart disease | 118808 | 132094 | 111780 | 166709 | 181869 | 151392 |
| Ischemic heart disease | 4903957 | 5023274 | 4806587 | 4026412 | 4144544 | 3934139 |
| Stroke | 3174188 | 3262513 | 3085563 | 2993104 | 3092111 | 2913101 |
| Hypertensive heart disease | 401747 | 434542 | 271578 | 523929 | 572203 | 372203 |
| Cardiomyopathy and myocarditis | 212930 | 228756 | 187422 | 155606 | 163326 | 149244 |
| Atrial fibrillation and flutter | 109971 | 124963 | 98219 | 177270 | 183825 | 170925 |
| Aortic aneurysm | 106344 | 113769 | 99955 | 60905 | 65291 | 58653 |
| Peripheral artery disease | 33785 | 61813 | 19572 | 36384 | 84565 | 16446 |
| Endocarditis | 39570 | 41876 | 37287 | 43820 | 53497 | 34539 |
| Non-rheumatic valvular heart disease | 63528 | 67697 | 52808 | 81331 | 86315 | 63869 |
| Other cardiovascular and circulatory diseases | 181507 | 203513 | 166208 | 179143 | 199117 | 165356 |
| DALYs Number | | | | | | |
| Cardiovascular diseases | 210268029 | 216021703 | 204242857 | 155601796 | 161530843 | 149718125 |
| Rheumatic heart disease | 4114459 | 4628634 | 3712342 | 5279101 | 5948308 | 4709554 |
| Ischemic heart disease | 105010694 | 107559509 | 102700766 | 65264654 | 67230249 | 63273733 |
| Stroke | 72179095 | 74959210 | 69350616 | 59872271 | 62994913 | 56681256 |
| Hypertensive heart disease | 7882381 | 8619687 | 5499027 | 8660821 | 9560878 | 6733748 |
| Cardiomyopathy and myocarditis | 6525926 | 7018606 | 5882722 | 3721154 | 4010641 | 3486155 |
| Atrial fibrillation and flutter | 2854170 | 3460593 | 2356088 | 3121835 | 3643027 | 2690845 |
| Aortic aneurysm | 2091233 | 2249363 | 1946020 | 948625 | 1049581 | 907062 |
| Peripheral artery disease | 758549 | 1193514 | 512922 | 673894 | 1149969 | 395430 |
| Endocarditis | 1159369 | 1231260 | 1090341 | 1068994 | 1221274 | 920264 |
| Non-rheumatic valvular heart disease | 1271865 | 1398630 | 1132193 | 1257312 | 1421119 | 1096884 |
| Other cardiovascular and circulatory diseases | 6420288 | 7414728 | 5622370 | 5733134 | 6750825 | 4945059 |

Table. S2. The Death number of cardiovascular disease and its 11 categories for different regions in 2017

| Regions | Cardiovascular diseases | Rheumatic heart disease | Ischemic heart disease | Stroke | Hypertensive heart disease | Cardiomyopathy and myocarditis | Atrial fibrillation and flutter | Aortic aneurysm | Peripheral artery disease | Endocarditis | Non-rheumatic valvular heart disease | Other cardiovascular and circulatory diseases |
| --- | --- | --- | --- | --- | --- | --- | --- | --- | --- | --- | --- | --- |
| Global | 17790949(18042674-17527068) | 285517(303298-266162) | 8930369(9138680-8790696) | 6167292(6327598-6044260) | 925675(994935-681438) | 368536(386921-341921) | 287241(304759-276355) | 167249(174146-159775) | 70169(123260-43181) | 83391(94325-74290) | 144860(150373-121827) | 360650(392918-338086) |
| East Asia | 4582561(4723877-4439766) | 72874(77046-69632) | 1827862(1890401-1770725) | 2208526(2286088-2139311) | 311953(343701-199418) | 32057(34504-30212) | 51760(54088-49453) | 17386(19225-15807) | 2532(3733-2056) | 7684(8038-7152) | 12157(15653-11435) | 37770(39899-32659) |
| Southeast Asia | 1341962(1398280-1295301) | 9932(10567-9370) | 555680(580475-532620) | 624441(651095-600193) | 68707(75539-54623) | 18779(21144-16835) | 14076(14994-13090) | 8998(10228-8093) | 622(807-467) | 10030(11084-8871) | 3494(4095-3198) | 27203(34327-23809) |
| Oceania | 31885(35899-28661) | 2168(2640-1722) | 15334(17526-13489) | 10417(12034-8987) | 1534(1876-999) | 631(809-511) | 158(176-143) | 236(310-181) | 7(9-5) | 346(442-260) | 179(227-146) | 876(1096-713) |
| Central Asia | 326641(338662-314917) | 3449(3695-3230) | 209672(218558-201416) | 85022(88235-81830) | 11381(12390-8736) | 8046(8877-6024) | 2552(2730-2395) | 1421(1481-1366) | 94(150-55) | 383(471-344) | 560(610-407) | 4061(4297-3812) |
| Central Europe | 653063(665061-641778) | 3010(3177-2884) | 349838(360429-342644) | 191530(197652-186711) | 34404(37578-24120) | 31335(32912-29158) | 10144(11104-9755) | 5772(6001-5517) | 4893(8798-2825) | 1301(1604-1152) | 5884(6234-4324) | 14952(16381-14175) |
| Eastern Europe | 1569210(1592671-1548813) | 5125(5384-4939) | 969015(993715-951967) | 437235(448234-429631) | 23708(25739-13329) | 76563(79672-68201) | 14875(15621-14158) | 10856(11190-10524) | 11787(20756-6798) | 2363(2725-1962) | 2291(2431-1634) | 15391(16033-14772) |
| High-income Asia Pacific | 441546(452623-429559) | 5624(5977-5387) | 179539(185151-173703) | 172255(177507-166659) | 15966(28910-13978) | 8969(9791-8527) | 14548(16324-13668) | 20565(21361-19542) | 1428(2495-752) | 4570(6929-3769) | 10824(11594-7272) | 7258(7615-6899) |
| Australasia | 62998(67318-59077) | 656(715-604) | 33667(36027-31368) | 14955(16140-13832) | 1418(1849-1143) | 1604(1999-1435) | 3977(4324-3535) | 1704(1846-1566) | 1515(3096-800) | 502(587-391) | 1941(2127-1578) | 1059(1159-968) |
| Western Europe | 1354433(1393265-1318828) | 17337(18853-16475) | 662226(707394-637966) | 330431(353582-318672) | 74727(83975-33798) | 43621(46219-38163) | 67016(78795-63651) | 30905(31987-29882) | 18597(33803-10316) | 15025(17683-11057) | 46288(49211-35863) | 48259(50947-46073) |
| Southern Latin America | 145601(155741-136672) | 3559(3914-3249) | 70410(75732-65615) | 37330(40038-34754) | 9306(10986-7330) | 5160(5989-4679) | 4332(4891-4025) | 3500(3814-3221) | 542(974-311) | 1919(2574-1475) | 2799(3201-2343) | 6744(7370-6106) |
| High-income North America | 983238(1000783-965143) | 11895(12360-11435) | 581610(597473-566264) | 190423(196482-184072) | 44683(47963-27958) | 31827(36197-30399) | 34532(37625-31832) | 14993(15481-14502) | 17421(35165-9177) | 10141(12738-8209) | 24323(25491-19166) | 21390(22163-20650) |
| Caribbean | 116124(122385-110055) | 1698(1986-1473) | 59059(62328-55895) | 34992(37333-32789) | 8139(9057-6865) | 2645(2904-2316) | 2209(2368-2092) | 1508(1646-1390) | 918(1620-561) | 668(804-589) | 997(1114-903) | 3290(3684-2999) |
| Andean Latin America | 62674(67097-58330) | 835(906-763) | 32409(34915-30078) | 18350(19718-16963) | 4331(4852-3557) | 757(835-693) | 2226(2393-2045) | 790(867-715) | 71(90-52) | 548(622-493) | 383(425-349) | 1974(2235-1777) |
| Central Latin America | 338616(349329-328241) | 1856(1958-1775) | 205117(213229-195835) | 79471(82526-75656) | 22050(30410-19975) | 4304(4577-3936) | 8575(9254-8087) | 3458(3653-3266) | 1370(2846-725) | 1757(2222-1518) | 2558(2750-1904) | 8101(8542-7681) |
| Tropical Latin America | 398103(402495-393752) | 2732(2847-2635) | 180722(184321-176381) | 126084(128777-123208) | 23642(31238-19691) | 18919(21839-17996) | 10284(10941-9644) | 10080(10468-9617) | 3758(7533-1868) | 3077(3886-2569) | 4079(4276-3208) | 14727(15207-14276) |
| North Africa and Middle East | 1123012(1160980-1087708) | 7115(9588-5990) | 709773(748432-677280) | 268625(285652-251232) | 74013(83077-43665) | 10505(11597-9667) | 8008(9293-7556) | 5674(7143-5177) | 1179(1668-928) | 3681(4025-3329) | 6303(6977-5746) | 28135(31552-20953) |
| South Asia | 3385169(3506002-3224254) | 119731(137210-102640) | 1918908(2000481-1828157) | 1019437(1067411-965026) | 124536(153367-94807) | 48508(61688-37197) | 27598(32857-22323) | 21694(25800-17970) | 1484(2320-636) | 10621(13662-9042) | 14684(16720-12200) | 77967(105227-62611) |
| Central Sub-Saharan Africa | 121790(134738-109700) | 3140(3704-2556) | 52917(60271-46362) | 41014(46684-35745) | 12877(19017-7466) | 3479(4992-2389) | 1210(1523-957) | 1188(1516-919) | 187(379-33) | 1047(1421-722) | 726(865-606) | 4004(6869-2644) |
| Eastern Sub-Saharan Africa | 304369(325071-286615) | 4809(5550-4068) | 123674(138053-111283) | 114245(125492-103067) | 31350(46107-17061) | 8548(9964-6383) | 3289(4287-2020) | 2815(3511-2047) | 445(959-81) | 2527(3349-1697) | 1919(2361-1606) | 10750(16632-7201) |
| Southern Sub-Saharan Africa | 104671(108921-101122) | 1975(2165-1811) | 44918(47020-43082) | 33740(35469-32347) | 11461(13246-10561) | 4284(4585-4064) | 1287(1350-1175) | 1207(1393-1121) | 862(1142-690) | 527(567-492) | 758(852-705) | 3651(3960-3140) |
| Western Sub-Saharan Africa | 343283(386378-308807) | 5997(6788-5253) | 148017(168534-132479) | 128768(145445-115866) | 15492(20901-9415) | 7993(9673-6519) | 4586(5469-3758) | 2499(3080-2063) | 457(987-148) | 4675(5554-3948) | 1711(2140-1342) | 23087(33069-17932) |
| World Bank High Income | 3337849(3396932-3288824) | 38750(40854-37541) | 1742723(1813996-1705715) | 829440(865360-811501) | 160278(171724-99574) | 107751(112260-102617) | 130546(148084-125381) | 74319(75993-72428) | 43536(83516-24455) | 32788(40549-25808) | 88836(93084-68182) | 88884(92360-86133) |
| World Bank Upper Middle Income | 7550851(7691823-7410923) | 88192(92258-84968) | 3475766(3545053-3413309) | 3056243(3140857-2983359) | 450801(487993-320241) | 148584(152890-137953) | 97358(101312-94409) | 51689(54549-49152) | 20718(34218-13774) | 22476(25313-20977) | 29599(32459-27485) | 109424(113001-102766) |
| World Bank Lower Middle Income | 6104616(6271582-5922188) | 141411(158661-124541) | 3363371(3463401-3261630) | 1975819(2049156-1902741) | 253700(290149-202573) | 96751(111598-82993) | 51803(57521-45570) | 35751(40648-31511) | 5258(7256-3271) | 23001(26148-20642) | 22476(25101-19624) | 135275(165491-117324) |
| World Bank Low Income | 713443(748511-679900) | 15769(17455-14519) | 313922(337649-292246) | 266888(284145-248869) | 55236(76416-34113) | 14717(17525-12130) | 6514(7864-4772) | 5092(6138-4068) | 561(1212-138) | 4925(6488-3516) | 3646(4274-3228) | 26173(36059-19874) |
| High-middle SDI | 4812251(4906187-4710147) | 35786(37467-34482) | 2485517(2532042-2439789) | 1726596(1774334-1680273) | 222081(240415-163874) | 126801(130548-115859) | 58589(61254-56766) | 34549(36187-33115) | 19270(32549-12217) | 13189(15163-12101) | 19000(20775-17457) | 70874(73464-67799) |
| High SDI | 3253770(3313618-3204021) | 37857(39847-36692) | 1694088(1761182-1659271) | 820929(853403-802492) | 152812(163557-99618) | 103566(107690-98222) | 128261(144665-123158) | 72685(74353-70796) | 41386(79341-23096) | 31370(38741-24637) | 85864(90088-65790) | 84950(88381-82312) |
| Low-middle SDI | 3064539(3181489-2944349) | 74204(84292-65298) | 1618139(1683816-1552777) | 1030351(1081572-983868) | 144096(163171-112636) | 46464(52568-41060) | 27577(30190-24587) | 18975(21344-17242) | 2244(3144-1354) | 12171(13635-10984) | 13366(15012-12036) | 76951(92222-67225) |
| Low SDI | 1610926(1678529-1533021) | 60141(69093-51043) | 775762(820501-737069) | 559178(591073-531719) | 94704(122603-67113) | 28498(36753-22253) | 13177(16081-10624) | 10860(13353-9166) | 828(1737-301) | 8339(11057-6549) | 7854(9195-6642) | 51585(66939-41422) |
| Middle SDI | 4965271(5079729-4842927) | 76133(80030-73200) | 2322276(2379738-2269736) | 1991334(2057004-1926578) | 306322(334480-213749) | 62474(66514-55942) | 58616(61610-55633) | 29780(32229-26939) | 6346(9670-4586) | 18120(19766-16643) | 18473(19839-16684) | 75395(81998-69762) |

Table. S3. The DALYs number of cardiovascular disease and its 11 categories for different regions in 2017.

| Regions | Cardiovascular diseases | Rheumatic heart disease | Ischemic heart disease | Stroke | Hypertensive heart disease | Cardiomyopathy and myocarditis | Atrial fibrillation and flutter | Aortic aneurysm | Peripheral artery disease | Endocarditis | Non-rheumatic valvular heart disease | Other cardiovascular and circulatory diseases |
| --- | --- | --- | --- | --- | --- | --- | --- | --- | --- | --- | --- | --- |
| Global | 365869825.2(376747292.3-355162644.2) | 9393559.871(10332599.73-8577116.766) | 170275348.1(174046939.4-167139660.3) | 132051366.5(137350169.2-126498599.6) | 16543201.61(17877866.8-12743136.28) | 10247080.53(10919714.37-9504712.446) | 5976005.199(7093786.033-5043626.673) | 3039857.777(3186409.803-2877173.644) | 1432442.912(2131537.105-969756.1555) | 2228363.287(2422500.994-2082112.113) | 2529177.497(2774571.278-2289317.037) | 12153421.97(13986047.6-10635141.97) |
| East Asia | 89290189(93337179-85585579) | 1909685.006(2136281.4-1728421.666) | 31536813.86(32678318.95-30439702.11) | 46475293.06(48994645.51-44022807.6) | 5084228.999(5615687.318-3448031.682) | 909128.6944(975845.8344-848057.2416) | 1207093.489(1452231.019-1004062.763) | 364349.7281(401375.2587-328389.009) | 174541.3699(281535.9431-99477.46114) | 177419.6844(186834.8401-163362.8944) | 247424.0507(306228.3158-225273.8175) | 1204211.442(1419086.77-1018478.493) |
| Southeast Asia | 32183307.72(33566008.16-30869983.05) | 482413.1516(547095.5594-431461.9674) | 12750704.81(13342554.91-12202403.22) | 14822771.02(15503456.64-14148969.79) | 1532519.686(1691758.36-1207845.371) | 559410.0341(629691.005-506578.34) | 328612.0329(393487.2139-277508.2527) | 178717.5429(204216.2415-160533.643) | 59172.50417(96926.80689-32362.8782) | 343744.3592(378895.1476-305846.2298) | 98132.40134(114630.0821-87301.60106) | 1027110.183(1250869.781-871888.9324) |
| Oceania | 1086369.9(1246988.7-961317.8) | 105704.13(128158.25-85106.59) | 473492.23(553008.66-407982.83) | 361465.72(424150.76-310033.35) | 47077.106(58986.465-29489.513) | 28639.13(36842.9-22623.588) | 4982.9965(5754.7331-4330.4794) | 6986.5543(9519.5908-4949.0428) | 728.63671(1175.1925-420.91899) | 14541.955(18822.007-10817.618) | 6773.2926(8913.8357-5183.7397) | 35978.189(45720.295-29092.526) |
| Central Asia | 6898911.95(7180517.39-6620262.37) | 142596.714(158129.698-129667.021) | 4058810.49(4256122.48-3880709) | 1930263.28(2024305.57-1834797.28) | 223825.954(249487.819-185315.341) | 273019.703(303786.984-201680.839) | 57729.2002(68747.7629-48807.0963) | 31776.7959(33387.5522-30365.0189) | 5297.63852(8154.64375-3164.03451) | 13707.2873(16619.9591-12363.7615) | 15748.1085(17711.9825-12675.5676) | 146136.779(162192.54-132479.175) |
| Central Europe | 10552776(10925907.3-10153968.1) | 61598.4205(64932.2841-58912.9852) | 5135059.8(5314591.18-5000794.76) | 3314124.71(3477000.67-3133839.99) | 503952.72(561757.804-389850.731) | 538778.566(571483.475-487841.098) | 210855.586(249748.281-177095.804) | 111007.945(115580.369-105780.394) | 79909.0523(128960.964-51315.1804) | 30161.8442(36185.4253-27184.2255) | 108381.265(120368.069-87303.6701) | 458946.048(532305.768-393275.248) |
| Eastern Europe | 28581552.31(29246779.2-27894853.49) | 135683.0141(142552.5997-130257.9132) | 16037271.87(16512219.99-15694031.87) | 8168961.673(8501681.347-7801775.316) | 426262.5306(460288.6464-278787.8401) | 2469584.359(2584003.133-2189495.483) | 320405.2779(381200.8463-270171.66) | 234696.6039(242933.0607-226445.5571) | 211607.2542(347763.2567-132265.9437) | 82567.38429(93606.76327-70516.56859) | 63415.72728(72622.44556-50164.83267) | 431096.6142(464055.8491-401984.8712) |
| High-income Asia Pacific | 6592529.93(7019305.79-6176810.93) | 57996.7374(61231.9543-55616.3309) | 2273795.72(2364631.69-2182379.66) | 2880114.91(3123437.14-2631402.98) | 216529.34(334965.521-180349.487) | 181870.441(204154.417-163785.315) | 214553.285(246524.24-187438.241) | 251426.134(263764.614-237576.11) | 31234.653(47032.2107-19465.375) | 58836.5231(84039.7271-49778.7718) | 128185.202(149567.267-104351.157) | 297986.984(380881.039-234618.718) |
| Australasia | 885444.36(955657.54-820153.48) | 10407.899(11348.428-9550.505) | 428464.88(463518.36-396591.21) | 213894.81(232124.88-195300.1) | 18439.563(24245.285-16006.004) | 39498.205(48992.442-35173.213) | 66048.521(78478.27-55216.206) | 23202.811(25329.442-21210.728) | 16241.968(30510.831-8880.7884) | 8192.1721(9463.4479-6697.6974) | 22477.937(25091.89-19605.746) | 38575.597(46956.418-31721.959) |
| Western Europe | 18496013.13(19503585.2-17556234.2) | 208337.1854(223278.1134-198568.5308) | 8314490.666(8733001.989-7990237.819) | 4695103.981(5030890.06-4352478.195) | 781541.273(863920.0517-521352.6965) | 680947.6179(736019.8577-628956.0034) | 1039397.154(1223000.763-890905.8511) | 427701.0622(443286.4674-411775.2689) | 250741.7705(406586.0563-154486.6529) | 203590.2897(240653.5603-163261.9365) | 565662.4208(625034.1505-484016.9301) | 1328499.713(1592450.302-1107322.539) |
| Southern Latin America | 2579182.81(2779287.15-2398940.23) | 72035.5042(81434.7079-63888.1847) | 1133468.27(1224873.38-1046270.57) | 734423.42(796019.689-675570.193) | 136540.399(165120.637-117880.743) | 110378.502(131349.344-100277.98) | 79579.6886(94872.5426-66913.3751) | 59525.9504(65188.7489-54462.8419) | 11079.0171(16912.555-7203.13597) | 35328.5456(46570.0153-29499.4883) | 46187.6505(52399.6175999999-40632.4545) | 160635.865(183559.156-141423.726) |
| High-income North America | 16928479.7(17689913.37-16186687.4) | 187020.878(194740.4308-179016.222) | 8688158.46(8950903.197-8442239.58) | 3905904.36(4277726.405-3538683.79) | 868604.12(941446.0493-536511.984) | 737763.785(848468.2843-693502.981) | 766779.281(920005.1813-635462.262) | 249767.296(258484.8392-240955.149) | 262396.893(476640.8358-154764.529) | 182347.151(225863.0119-155742.284) | 331773.466(376540.8964-290897.86) | 747964.054(871363.2668-650899.694) |
| Caribbean | 2373873.79(2528221.93-2233450.27) | 71923.0669(85803.2974-61302.4829) | 1102217.74(1181010.95-1033831.66) | 725513.742(785172.305-675544.99) | 161467.185(182215.089-140574.873) | 77543.6824(87066.0034-66596.2661) | 45080.9669(53077.6562-38346.2608) | 26927.1533(29507.0654-24684.3974) | 15026.733(23385.4922-9854.07544) | 20942.3123(24706.2754-18311.788) | 22602.6372(25464.6726-20440.0841) | 104628.572(119500.748-91693.4294) |
| Andean Latin America | 1304771.3(1404790.2-1202643.3) | 42563.351(52119.43-35003.265) | 585682.29(635679.02-539749.3) | 398410.86(431145.26-365023.89) | 77971.489(87020.162-64259.358) | 26925.235(30114.874-24388.707) | 45923.121(54527.759-38168.846) | 15241.364(16929.894-13637.92) | 3691.4175(5871.8578-2178.9025) | 16038.146(18357.446-14267.313) | 10627.069(11916.675-9466.1953) | 81696.976(97060.08-68873.325) |
| Central Latin America | 6843413.47(7154530.28-6530677.12) | 87759.4921(103972.785-75624.9101) | 3761320.16(3921089.87-3602102.06) | 1678882.13(1759638.85-1599304.37) | 390749.199(513922.246-349939.941) | 146690.87(156153.108-138066.492) | 205677.467(248907.368-170816.868) | 66743.0688(70817.5803-62760.3501) | 33490.8599(54583.2473-20108.1856) | 59919.364(76438.1519-53572.8893) | 65113.9602(70821.2771-51652.7924) | 347066.897(412372.943-296743.372) |
| Tropical Latin America | 8682885.68(8953653.07-8416825.53) | 166051.47(203502.121-137381.155) | 3773723.59(3864074.07-3669859.27) | 2662717.44(2756370.84-2578218.08) | 445405.823(580936.776-392316.077) | 493638.53(560011.092-468903.237) | 232241.967(278609.927-192597.669) | 213429.055(222230.759-203493.539) | 69881.1929(124018.953-40778.1604999999) | 91030.0797(116380.148-79186.1801) | 92146.7866(98356.7515-78029.5285) | 442619.748(491372.919-403536.248) |
| North Africa and Middle East | 26270969.39(27479231.94-25170861.19) | 396918.9831(494338.4901-328745.2877) | 15591388.44(16458496.72-14772624.39) | 6615965.247(7073044.247-6167119.796) | 1446198.669(1621497.394-906945.6097) | 439775.1896(486844.7708-399160.2259) | 187440.3754(223775.812-156783.0768) | 127841.3109(159705.8248-116621.699) | 42747.71442(62521.98961-29033.72659) | 118186.4332(130524.1177-105467.0064) | 169845.9743(191989.2288-152001.7176) | 1134661.053(1320930.737-889569.4043) |
| South Asia | 83458337.85(86408582.02-80091339.13) | 4218212.369(4773761.484-3659606.697) | 46180775.47(47965579.76-44281899.82) | 24337648.22(25448470.19-23173522.21) | 2584413.434(3201811.154-1971666.208) | 1539449.064(1952588.413-1204483.245) | 728796.0384(889792.5035-593761.9417) | 466137.062(555965.3716-388157.116) | 99139.05052(159519.1992-56369.1826) | 325631.8964(415742.6017-276915.6676) | 372441.717(426160.4568-306597.0394) | 2605693.529(3338714.955-2155468.444) |
| Central Sub-Saharan Africa | 3238833.41(3580570.17-2902979.96) | 168471.753(199227.341-138344.663) | 1272272.81(1446260.6-1106494.38) | 1080403.89(1225254.13-942358.52) | 287936.088(407273.305-165936.015) | 152604.94(208762.516-110066.164) | 28842.1418(35099.2984-23338.661) | 29927.3003(39060.7242-23174.7546) | 6643.3423(11038.8636-2747.62698) | 45268.7804(62895.0703-32864.0121999999) | 20542.409(24824.7796-17085.9649) | 145919.947(249116.393-100129.978) |
| Eastern Sub-Saharan Africa | 8184186.93(8707633.5-7687775.03) | 373523.37(456698.38-303684.475) | 2980517.44(3275919.78-2714447.12) | 2964840.52(3204053.9-2707005.9) | 687009.448(971828.524-383245.078) | 401012.653(463158.602-317640.2) | 80131.2424(101042.034-59229.6093) | 69818.2905(87192.338-51168.0056) | 18195.5386(29135.663-8180.94309) | 118490.626(157619.363-82538.8342) | 56628.9475(67019.2002-48895.8405) | 434018.855(654991.601-312330.247) |
| Southern Sub-Saharan Africa | 2404275.47(2515860.78-2307500.77) | 108809.155(128299.169-93638.3454) | 944129.697(989311.195-902032.382) | 759381.039(800540.043-724374.318) | 227011.527(267593.88-211386.15) | 123212.12(133473.865-114830.893) | 29926.6312(35461.2016-25318.8342) | 26749.8864(30411.377-24632.8364) | 21677.6805(28174.0439-17117.569) | 19981.1574(21733.7426-18383.5665) | 20492.3392(23873.846-18772.0629) | 122904.239(137579.808-99235.7748) |
| Western Sub-Saharan Africa | 9033520.71(10091098-8169208.09) | 385848.216(463893.844-317282.833) | 3252789.47(3713214.99-2908265.98) | 3325282.41(3723269.46-3030455.73) | 395517.062(536079.76-242918.401) | 317209.204(372659.683-265004.861) | 95908.7352(116191.811-79081.8839) | 57884.8627(70508.6922-47825.3267) | 18998.6253(31041.8345-10265.1323) | 262437.296(320321.066-210707.67) | 64574.135(82166.3471-49948.3382) | 857070.69(1193608.86-683510.894) |
| High HDI | 51893088.42(54390895.25-49495466.21) | 538727.1727(562929.1732-523105.5362) | 24319414.71(25093561.34-23742256.81) | 14173252.89(15228172.4-13066188.26) | 2273524.309(2485671.201-1612663.435) | 2075206.057(2236354.897-1962860.999) | 2299251.598(2711626.452-1949452.822) | 1071938.919(1098911.164-1042781.048) | 637397.8894(1074358.033-411359.6534) | 512789.6372(621581.5629-431693.0836) | 1152523.837(1284251.545-994271.8939) | 2839061.396(3394688.693-2386671.914) |
| Upper Middle HDI | 147382576(152671562.4-142469142.7) | 2574926.075(2907032.876-2307941.081) | 61192320.51(62709458-59853951.43) | 63137257.34(66222139.25-60118310.27) | 7521299.896(8150239.335-5690966.762) | 4342568.183(4487887.312-3953321.977) | 2211111.919(2645250.626-1847737.567) | 1056654.894(1114586.241-1002225.026) | 533504.4408(778839.8355-366316.9992) | 618176.855(694193.7094-576192.5852) | 650713.5309(717929.6488-601664.3311) | 3544042.361(4062857.199-3135949.292) |
| Lower Middle HDI | 146228939.5(150583390.6-141476224.3) | 5373200.555(5999836.425-4784326.766) | 76722349.65(79033372.11-74460473.83) | 47064584.25(48838881.48-45233382.99) | 5426792.634(6244634.35-4274780.897) | 3155984.062(3627266.341-2746273.473) | 1284736.77(1550654.165-1068032.133) | 778342.0317(887491.5477-692252.0451) | 227657.6823(339016.5986-144051.5394) | 847664.9409(933809.0209-771138.7679) | 609365.2578(689852.2154-527673.2647) | 4738261.668(5600477.442-4103138.638) |
| Low HDI | 18689897.1(19579851.9-17727708) | 866064.898(995059.755-754263.615) | 7425437.93(7932349.01-6971609.62) | 6849031.92(7206397-6419284.6) | 1226647.05(1632952.24-770553.745) | 652185.116(769001.126-540213.986) | 157811.011(191869.638-125475.149) | 124850.058(154388.388-100602.341) | 30096.3397(47623.5035-15467.4455) | 244463.978(326007.214-179841.37) | 110485.088(132277.102-95826.1901) | 1002823.71(1372819.14-781383.871) |
| High-middle SDI | 91958804(95088510-88907567) | 1016494(1135352-928671) | 43092169(44054875-42107958) | 35123163(36985332-33366160) | 3621775(3926007-2881558) | 3749188(3888466-3384885) | 1280292(1525857-1078646) | 709828(742498-677798) | 407227(604408-278077) | 351953(396311-325769) | 400381(443759-368434) | 2206335(2504154-1973702) |
| High SDI | 49967340(52340627-47668993) | 513633(535357-499196) | 23121439(23858636-22559867) | 14014905(15097414-12898819) | 2147213(2351964-1569279) | 1965202(2120852-1859392) | 2259843(2665974-1915677) | 1041693(1068815-1012521) | 610755(1022811-394005) | 480929(583231-402978) | 1103370(1231739-950093) | 2708356(3247775-2270782) |
| Low-middle SDI | 74906150(77777647-72042068) | 2845259(3189064-2535789) | 37904606(39504480-36397813) | 24783260(25971166-23666936) | 3018975(3473784-2351429) | 1598060(1790072-1438151) | 672536(804471-564000) | 421055(475980-378610) | 108591(163195-66863) | 477912(531697-435046) | 359891(412135-317559) | 2716004(3157835-2363758) |
| Low SDI | 41940719(43657094-40000326) | 2500302(2836006-2172067) | 19110400(20030488-18267765) | 13994563(14672180-13333510) | 2087467(2615751-1458872) | 1122263(1408722-895071) | 354810(432596-287893) | 250787(313401-209434) | 55875(88543-31265) | 365308(478783-289239) | 224076(269952-190019) | 1874867(2314031-1532987) |
| Middle SDI | 105421489(109007593-101963074) | 2477230(2758348-2253683) | 46430908(47696956-45224070) | 43308235(45203779-41366802) | 5572834(6076147-4116264) | 1791230(1931366-1590633) | 1385430(1666062-1159365) | 608422(660431-548591) | 246209(367857-157899) | 546993(600133-499523) | 435369(481017-392711) | 2618627(3022647-2278733) |

Table.S4. The ASDR of cardiovascular disease and its 11 categories in 195 countries and territories in 2017.

|  | Cardiovascular diseases | Rheumatic heart disease | Ischemic heart disease | Stroke | Hypertensive heart disease | Cardiomyopathy and myocarditis | Atrial fibrillation and flutter | Aortic aneurysm | Peripheral artery disease | Endocarditis | Non-rheumatic valvular heart disease | Other cardiovascular and circulatory diseases |
| --- | --- | --- | --- | --- | --- | --- | --- | --- | --- | --- | --- | --- |
| Uzbekistan | 724.42 | 5.44 | 534.24 | 149.14 | 19.83 | 1.89 | 6.23 | 1.30 | 0.16 | 0.56 | 1.33 | 4.29 |
| Afghanistan | 597.03 | 6.87 | 360.01 | 165.80 | 43.17 | 2.40 | 2.31 | 1.22 | 0.03 | 0.43 | 1.60 | 13.18 |
| Papua New Guinea | 561.49 | 30.56 | 267.48 | 198.15 | 27.74 | 8.29 | 4.06 | 4.19 | 0.08 | 4.48 | 2.90 | 13.56 |
| Azerbaijan | 559.81 | 2.77 | 381.76 | 135.10 | 9.56 | 12.29 | 5.35 | 2.02 | 0.12 | 0.71 | 0.52 | 9.62 |
| Marshall Islands | 557.79 | 16.28 | 261.90 | 201.20 | 30.89 | 11.71 | 6.59 | 5.26 | 0.40 | 5.62 | 3.90 | 14.05 |
| Vanuatu | 546.30 | 20.09 | 283.33 | 176.76 | 25.54 | 8.93 | 5.68 | 4.10 | 0.13 | 2.71 | 3.39 | 15.63 |
| Ukraine | 539.85 | 1.51 | 386.14 | 109.90 | 1.69 | 24.90 | 5.25 | 2.56 | 3.02 | 0.67 | 0.42 | 3.79 |
| Turkmenistan | 536.78 | 2.86 | 339.30 | 148.85 | 17.03 | 12.21 | 5.74 | 2.36 | 0.28 | 0.58 | 0.36 | 7.22 |
| Egypt | 525.43 | 1.85 | 350.68 | 120.83 | 32.52 | 2.81 | 3.17 | 1.44 | 0.17 | 0.63 | 1.80 | 9.53 |
| Georgia | 496.22 | 8.83 | 249.46 | 167.07 | 46.22 | 4.97 | 7.02 | 2.97 | 0.19 | 0.70 | 1.89 | 6.89 |
| Yemen | 495.00 | 4.22 | 300.41 | 135.10 | 34.58 | 2.61 | 2.66 | 1.32 | 0.04 | 0.70 | 1.61 | 11.76 |
| Kazakhstan | 466.79 | 2.85 | 275.19 | 135.34 | 10.67 | 28.58 | 4.62 | 1.93 | 0.14 | 0.50 | 0.41 | 6.57 |
| Mongolia | 460.04 | 5.31 | 248.23 | 177.86 | 9.01 | 8.55 | 4.04 | 0.94 | 0.09 | 0.48 | 1.66 | 3.88 |
| Solomon Islands | 459.78 | 15.02 | 236.20 | 164.14 | 19.42 | 5.35 | 3.62 | 2.50 | 0.11 | 2.49 | 2.07 | 8.87 |
| Federated States of Micronesia | 454.34 | 13.00 | 223.37 | 156.33 | 23.88 | 8.53 | 6.06 | 4.12 | 0.34 | 4.18 | 3.30 | 11.23 |
| Belarus | 443.13 | 1.68 | 318.00 | 95.66 | 6.19 | 9.21 | 3.92 | 2.48 | 1.04 | 0.55 | 0.25 | 4.16 |
| Serbia | 439.42 | 1.06 | 208.23 | 167.93 | 18.05 | 17.95 | 6.18 | 4.03 | 0.76 | 0.61 | 5.28 | 9.33 |
| Kyrgyzstan | 436.36 | 3.18 | 293.14 | 115.06 | 10.34 | 6.53 | 3.02 | 0.78 | 0.43 | 0.26 | 0.29 | 3.32 |
| Central African Republic | 435.73 | 11.73 | 188.86 | 156.48 | 46.62 | 7.43 | 4.73 | 3.57 | 0.36 | 2.16 | 2.09 | 11.69 |
| Kiribati | 434.66 | 9.97 | 182.90 | 182.00 | 22.28 | 8.43 | 3.72 | 1.61 | 0.22 | 6.50 | 3.29 | 13.74 |
| Sudan | 431.39 | 3.30 | 263.13 | 114.86 | 30.28 | 2.69 | 2.55 | 1.10 | 0.07 | 0.59 | 1.46 | 11.36 |
| Russian Federation | 431.30 | 1.56 | 240.95 | 135.32 | 7.47 | 27.93 | 4.03 | 3.49 | 3.83 | 0.91 | 0.78 | 5.01 |
| Haiti | 430.55 | 12.23 | 185.39 | 153.45 | 37.39 | 9.20 | 5.68 | 3.69 | 0.50 | 2.31 | 3.28 | 17.43 |
| Tajikistan | 427.70 | 2.87 | 268.09 | 111.94 | 32.53 | 0.96 | 3.73 | 1.89 | 0.07 | 0.40 | 0.70 | 4.51 |
| Bulgaria | 424.69 | 1.96 | 220.41 | 135.87 | 36.05 | 4.91 | 4.75 | 2.15 | 0.22 | 1.02 | 1.00 | 16.34 |
| Pakistan | 423.03 | 5.95 | 235.54 | 133.72 | 18.16 | 5.81 | 6.31 | 3.24 | 0.24 | 1.00 | 2.19 | 10.89 |
| Morocco | 419.15 | 1.90 | 276.00 | 99.80 | 24.80 | 2.31 | 2.78 | 1.13 | 0.09 | 0.53 | 1.40 | 8.40 |
| Fiji | 412.82 | 11.56 | 226.65 | 92.13 | 30.53 | 9.51 | 5.00 | 5.76 | 0.25 | 8.13 | 3.09 | 20.23 |
| Moldova | 408.50 | 1.63 | 267.90 | 101.33 | 23.67 | 6.29 | 2.76 | 1.26 | 0.56 | 0.48 | 0.31 | 2.31 |
| Madagascar | 405.99 | 6.32 | 136.27 | 181.95 | 46.50 | 7.65 | 5.54 | 3.64 | 0.36 | 2.74 | 2.43 | 12.59 |
| Lesotho | 405.13 | 7.75 | 146.83 | 172.94 | 43.86 | 11.33 | 3.82 | 2.51 | 1.01 | 1.70 | 2.11 | 11.28 |
| Montenegro | 387.30 | 1.26 | 149.60 | 185.15 | 2.24 | 20.57 | 13.89 | 7.31 | 0.22 | 0.41 | 0.70 | 5.94 |
| Guinea-Bissau | 382.47 | 7.33 | 173.82 | 148.10 | 17.28 | 4.86 | 5.39 | 2.34 | 0.28 | 2.45 | 1.36 | 19.26 |
| Syria | 376.26 | 1.40 | 284.49 | 68.10 | 4.09 | 2.92 | 3.16 | 1.70 | 0.11 | 1.18 | 3.43 | 5.69 |
| Guyana | 373.16 | 3.42 | 166.62 | 127.47 | 39.79 | 10.75 | 4.89 | 3.20 | 0.75 | 3.12 | 3.38 | 9.76 |
| Romania | 370.95 | 1.36 | 174.14 | 125.25 | 30.12 | 23.28 | 3.67 | 2.08 | 1.80 | 0.47 | 1.68 | 7.09 |
| Philippines | 370.44 | 3.65 | 183.90 | 118.97 | 42.57 | 9.26 | 3.81 | 2.15 | 0.27 | 1.53 | 0.47 | 3.86 |
| Laos | 368.11 | 4.07 | 156.03 | 169.23 | 17.57 | 4.50 | 3.62 | 1.94 | 0.09 | 1.85 | 0.89 | 8.32 |
| Somalia | 365.77 | 5.30 | 162.56 | 136.15 | 38.33 | 4.67 | 4.20 | 2.41 | 0.29 | 1.18 | 1.68 | 9.01 |
| Latvia | 350.06 | 1.31 | 198.03 | 100.12 | 14.02 | 24.66 | 3.90 | 2.13 | 0.71 | 0.59 | 1.10 | 3.49 |
| Samoa | 348.98 | 7.71 | 173.69 | 116.56 | 19.08 | 7.21 | 5.83 | 3.14 | 0.32 | 3.21 | 2.93 | 9.31 |
| Congo | 344.09 | 5.12 | 159.33 | 109.92 | 36.15 | 7.78 | 6.24 | 3.81 | 1.36 | 2.40 | 2.20 | 9.78 |
| Lithuania | 342.99 | 1.73 | 233.16 | 74.66 | 7.33 | 10.59 | 3.98 | 2.60 | 1.26 | 0.75 | 0.91 | 6.01 |
| Indonesia | 342.86 | 1.01 | 131.51 | 178.32 | 14.70 | 3.54 | 3.90 | 1.76 | 0.18 | 1.31 | 0.79 | 5.84 |
| Libya | 341.86 | 1.02 | 234.17 | 71.12 | 18.89 | 2.88 | 2.73 | 1.07 | 0.33 | 0.85 | 1.33 | 7.45 |
| Armenia | 341.01 | 3.31 | 236.55 | 64.51 | 15.08 | 4.45 | 4.05 | 8.26 | 0.17 | 0.25 | 0.58 | 3.80 |
| Guinea | 336.72 | 5.90 | 157.44 | 130.91 | 13.38 | 4.11 | 4.45 | 2.03 | 0.15 | 1.94 | 1.06 | 15.36 |
| Timor-Leste | 335.35 | 3.55 | 137.61 | 161.46 | 14.65 | 3.95 | 3.54 | 1.51 | 0.06 | 1.27 | 0.84 | 6.93 |
| Swaziland | 333.44 | 5.00 | 140.95 | 126.30 | 31.76 | 9.75 | 3.52 | 2.63 | 1.35 | 1.62 | 1.78 | 8.78 |
| The Gambia | 331.43 | 4.07 | 170.31 | 114.93 | 12.72 | 4.19 | 5.07 | 2.02 | 0.29 | 1.56 | 1.08 | 15.20 |
| Mozambique | 329.94 | 3.37 | 111.10 | 153.35 | 37.29 | 4.83 | 4.32 | 3.19 | 0.53 | 1.46 | 1.74 | 8.75 |
| Bosnia and Herzegovina | 329.63 | 1.11 | 164.97 | 123.52 | 4.73 | 12.82 | 5.56 | 3.13 | 0.17 | 0.42 | 2.62 | 10.59 |
| Sierra Leone | 325.72 | 4.77 | 161.89 | 116.22 | 12.98 | 4.12 | 4.15 | 1.90 | 0.15 | 1.90 | 1.03 | 16.61 |
| Macedonia | 322.69 | 1.01 | 125.91 | 149.79 | 20.20 | 12.44 | 4.39 | 1.67 | 0.14 | 0.34 | 0.56 | 6.25 |
| North Korea | 321.68 | 7.19 | 111.15 | 171.72 | 19.32 | 2.35 | 3.50 | 1.17 | 0.07 | 0.53 | 1.09 | 3.60 |
| Tunisia | 318.99 | 0.88 | 205.05 | 75.06 | 22.31 | 2.19 | 3.19 | 1.04 | 0.18 | 0.64 | 1.35 | 7.10 |
| Democratic Republic of the Congo | 318.95 | 5.63 | 143.08 | 107.58 | 37.29 | 5.67 | 4.47 | 2.62 | 0.42 | 1.80 | 1.69 | 8.69 |
| United Arab Emirates | 317.84 | 3.81 | 179.50 | 93.25 | 27.39 | 3.75 | 3.40 | 2.07 | 0.46 | 1.20 | 1.73 | 1.29 |
| Eritrea | 311.11 | 4.33 | 121.46 | 117.34 | 40.19 | 6.11 | 5.43 | 2.47 | 0.46 | 2.35 | 1.88 | 9.08 |
| Guam | 310.50 | 3.37 | 202.44 | 61.90 | 18.31 | 5.37 | 3.93 | 3.61 | 0.57 | 3.91 | 2.12 | 4.95 |
| Zimbabwe | 307.85 | 7.13 | 164.80 | 82.84 | 13.59 | 11.28 | 3.44 | 2.92 | 0.10 | 0.45 | 1.58 | 19.72 |
| Albania | 304.20 | 1.36 | 146.26 | 121.23 | 3.23 | 9.12 | 3.95 | 1.07 | 0.11 | 0.81 | 1.52 | 15.52 |
| Cote d'Ivoire | 303.74 | 4.60 | 148.86 | 109.24 | 11.95 | 4.55 | 4.63 | 1.95 | 0.28 | 1.74 | 1.08 | 14.85 |
| Ghana | 298.25 | 2.32 | 131.60 | 122.89 | 10.27 | 8.83 | 3.76 | 1.75 | 0.16 | 2.20 | 0.93 | 13.53 |
| Bangladesh | 298.00 | 2.93 | 117.40 | 153.00 | 10.13 | 3.00 | 2.82 | 1.59 | 0.12 | 0.58 | 1.05 | 5.36 |
| Burundi | 293.07 | 3.34 | 125.82 | 104.30 | 38.25 | 4.41 | 3.47 | 1.99 | 0.59 | 1.11 | 1.34 | 8.45 |
| Slovakia | 287.96 | 1.08 | 193.48 | 58.63 | 10.90 | 5.37 | 5.13 | 1.88 | 0.79 | 0.61 | 1.90 | 8.17 |
| American Samoa | 283.75 | 6.76 | 139.09 | 89.60 | 12.40 | 10.11 | 6.18 | 2.42 | 0.52 | 5.40 | 2.17 | 9.10 |
| India | 282.28 | 10.44 | 164.68 | 77.42 | 11.86 | 3.86 | 3.28 | 1.82 | 0.16 | 0.88 | 1.28 | 6.59 |
| Chad | 281.00 | 6.04 | 124.76 | 109.46 | 12.75 | 3.72 | 4.03 | 1.71 | 0.09 | 1.82 | 0.89 | 15.71 |
| South Sudan | 280.78 | 4.16 | 121.03 | 106.24 | 28.13 | 4.57 | 3.79 | 2.15 | 0.32 | 1.30 | 1.50 | 7.58 |
| Togo | 280.03 | 4.19 | 135.30 | 102.76 | 11.39 | 3.61 | 4.27 | 1.89 | 0.20 | 1.86 | 0.97 | 13.60 |
| Algeria | 278.36 | 0.95 | 172.38 | 67.76 | 21.53 | 2.44 | 2.91 | 0.93 | 0.23 | 0.66 | 1.30 | 7.28 |
| Hungary | 278.30 | 1.38 | 165.45 | 57.12 | 22.33 | 11.11 | 3.76 | 2.55 | 4.52 | 0.95 | 4.80 | 4.32 |
| Angola | 276.04 | 5.14 | 115.05 | 94.08 | 32.96 | 6.70 | 5.26 | 3.14 | 0.77 | 1.89 | 1.98 | 9.06 |
| Virgin Islands, U.S. | 273.67 | 1.50 | 154.63 | 50.93 | 24.89 | 11.60 | 6.65 | 3.26 | 3.49 | 3.02 | 3.26 | 10.42 |
| Liberia | 272.51 | 3.75 | 138.71 | 94.56 | 10.63 | 3.60 | 4.24 | 1.70 | 0.17 | 1.32 | 0.88 | 12.96 |
| Cambodia | 270.89 | 2.71 | 81.39 | 153.99 | 15.96 | 3.49 | 3.14 | 1.45 | 0.08 | 1.55 | 0.70 | 6.44 |
| Iran | 270.31 | 1.73 | 159.53 | 61.28 | 29.53 | 2.57 | 3.20 | 0.97 | 0.30 | 0.75 | 1.50 | 8.95 |
| Sao Tome and Principe | 270.11 | 3.89 | 121.64 | 102.74 | 4.71 | 4.03 | 4.84 | 1.78 | 0.30 | 1.41 | 1.03 | 23.75 |
| Burkina Faso | 269.05 | 3.17 | 135.87 | 79.03 | 17.01 | 4.59 | 5.05 | 1.62 | 0.15 | 2.02 | 1.16 | 19.38 |
| Mali | 268.02 | 5.10 | 114.51 | 107.12 | 11.98 | 4.15 | 5.23 | 1.76 | 0.12 | 2.02 | 0.94 | 15.09 |
| Dominican Republic | 266.65 | 2.00 | 151.32 | 78.47 | 16.07 | 2.99 | 4.36 | 2.02 | 0.25 | 1.19 | 1.94 | 6.04 |
| Lebanon | 266.59 | 0.49 | 192.17 | 41.40 | 18.30 | 1.92 | 3.16 | 1.12 | 0.22 | 0.70 | 1.31 | 5.81 |
| Oman | 266.34 | 0.50 | 183.45 | 57.27 | 3.48 | 2.78 | 4.18 | 1.89 | 0.34 | 1.01 | 1.60 | 9.84 |
| Palestine | 265.91 | 0.68 | 152.98 | 81.50 | 13.04 | 3.12 | 3.36 | 1.60 | 0.08 | 1.78 | 1.15 | 6.63 |
| China | 261.90 | 4.03 | 107.22 | 122.41 | 18.63 | 1.98 | 3.41 | 0.88 | 0.15 | 0.42 | 0.68 | 2.09 |
| Comoros | 261.52 | 2.55 | 130.54 | 86.57 | 23.00 | 3.91 | 3.75 | 2.29 | 0.35 | 1.12 | 1.36 | 6.07 |
| Malaysia | 260.94 | 1.40 | 149.77 | 83.74 | 2.36 | 2.97 | 3.79 | 3.97 | 0.21 | 3.33 | 0.78 | 8.65 |
| Nepal | 260.80 | 6.99 | 157.30 | 74.12 | 8.76 | 2.55 | 2.56 | 1.52 | 0.07 | 0.39 | 1.06 | 5.47 |
| Gabon | 259.97 | 3.04 | 121.25 | 77.25 | 27.59 | 7.23 | 6.03 | 3.67 | 2.02 | 1.87 | 2.01 | 8.01 |
| Saudi Arabia | 259.54 | 0.70 | 167.71 | 68.88 | 2.42 | 5.54 | 3.78 | 1.53 | 0.25 | 2.27 | 0.93 | 5.53 |
| Suriname | 258.31 | 2.14 | 107.79 | 102.12 | 20.29 | 6.68 | 5.14 | 2.48 | 0.22 | 1.44 | 2.93 | 7.09 |
| Djibouti | 258.04 | 2.20 | 127.98 | 84.22 | 22.70 | 4.78 | 4.19 | 2.43 | 0.78 | 1.18 | 1.56 | 6.02 |
| Estonia | 255.57 | 1.14 | 141.09 | 37.09 | 51.93 | 9.74 | 4.42 | 2.81 | 0.29 | 0.79 | 2.48 | 3.80 |
| Croatia | 253.78 | 1.22 | 142.41 | 72.23 | 14.53 | 9.01 | 3.15 | 3.47 | 1.18 | 0.41 | 3.36 | 2.80 |
| Saint Vincent and the Grenadines | 252.68 | 2.48 | 113.19 | 76.08 | 29.78 | 5.15 | 4.75 | 2.85 | 1.41 | 1.69 | 2.24 | 13.05 |
| Vietnam | 245.46 | 2.28 | 80.16 | 135.95 | 11.51 | 3.28 | 3.50 | 1.51 | 0.10 | 1.20 | 0.64 | 5.34 |
| Cameroon | 244.66 | 3.31 | 101.03 | 94.66 | 12.94 | 5.03 | 5.13 | 1.82 | 0.46 | 2.30 | 1.17 | 16.80 |
| Grenada | 243.96 | 3.89 | 103.93 | 78.11 | 19.16 | 9.53 | 5.36 | 4.62 | 2.01 | 3.17 | 2.72 | 11.47 |
| Namibia | 243.81 | 3.25 | 101.43 | 91.66 | 22.93 | 7.97 | 3.12 | 2.45 | 0.67 | 1.09 | 1.42 | 7.83 |
| Seychelles | 242.65 | 1.45 | 95.60 | 56.13 | 59.85 | 9.51 | 3.94 | 1.99 | 0.54 | 1.46 | 0.99 | 11.19 |
| Senegal | 241.22 | 3.43 | 119.89 | 87.13 | 9.20 | 3.08 | 3.94 | 1.31 | 0.16 | 1.18 | 0.79 | 11.11 |
| Honduras | 240.21 | 0.48 | 152.56 | 56.49 | 15.05 | 3.71 | 4.01 | 1.25 | 0.25 | 0.41 | 0.79 | 5.21 |
| Niger | 238.34 | 5.78 | 98.18 | 98.85 | 11.83 | 3.14 | 3.74 | 1.33 | 0.07 | 1.40 | 0.80 | 13.23 |
| Botswana | 237.37 | 2.58 | 103.36 | 85.43 | 22.05 | 8.00 | 3.11 | 2.35 | 1.61 | 1.01 | 1.37 | 6.49 |
| The Bahamas | 235.95 | 1.85 | 90.04 | 60.29 | 47.98 | 9.07 | 5.70 | 3.64 | 2.34 | 2.11 | 2.32 | 10.61 |
| Benin | 235.85 | 4.24 | 98.67 | 93.77 | 11.76 | 3.87 | 4.83 | 1.69 | 0.21 | 1.88 | 0.99 | 13.93 |
| Zambia | 234.50 | 2.20 | 107.73 | 81.17 | 23.39 | 4.32 | 3.78 | 2.13 | 0.53 | 1.36 | 1.41 | 6.49 |
| Mauritania | 232.35 | 2.94 | 116.21 | 79.20 | 9.07 | 3.95 | 5.01 | 1.87 | 0.43 | 1.83 | 0.89 | 10.94 |
| Trinidad and Tobago | 228.47 | 1.43 | 122.05 | 64.77 | 16.50 | 5.32 | 4.10 | 4.18 | 0.69 | 1.41 | 1.32 | 6.68 |
| Tonga | 227.49 | 3.29 | 122.88 | 72.76 | 7.58 | 2.01 | 4.59 | 1.98 | 0.53 | 2.49 | 2.28 | 7.12 |
| Czech Republic | 227.48 | 1.37 | 147.12 | 48.33 | 5.76 | 4.69 | 3.96 | 2.75 | 1.21 | 0.86 | 3.19 | 8.26 |
| Dominica | 227.38 | 2.86 | 77.95 | 59.66 | 33.99 | 19.74 | 6.21 | 4.37 | 1.51 | 2.49 | 2.47 | 16.13 |
| Malawi | 227.35 | 2.42 | 103.72 | 79.40 | 24.80 | 3.39 | 3.19 | 2.03 | 0.36 | 0.82 | 1.25 | 5.97 |
| Poland | 227.33 | 1.57 | 130.81 | 53.16 | 5.64 | 17.79 | 4.81 | 2.92 | 3.42 | 0.64 | 2.12 | 4.48 |
| Mauritius | 224.64 | 1.53 | 117.14 | 68.08 | 23.60 | 3.49 | 3.80 | 1.09 | 0.30 | 1.14 | 0.72 | 3.78 |
| Kenya | 218.64 | 2.13 | 88.98 | 84.53 | 22.75 | 4.49 | 3.94 | 1.99 | 0.49 | 1.38 | 1.43 | 6.52 |
| Iraq | 218.61 | 1.10 | 129.73 | 67.15 | 7.48 | 3.45 | 2.30 | 1.46 | 0.07 | 0.34 | 1.17 | 4.36 |
| Tanzania | 217.29 | 1.82 | 102.45 | 65.65 | 26.07 | 4.55 | 3.87 | 2.49 | 0.45 | 1.21 | 1.44 | 7.27 |
| Bhutan | 217.07 | 5.40 | 120.38 | 63.39 | 9.77 | 3.71 | 3.79 | 2.53 | 0.19 | 0.65 | 1.27 | 5.98 |
| Uganda | 213.33 | 2.18 | 86.58 | 77.83 | 26.73 | 4.10 | 3.81 | 1.72 | 0.37 | 1.03 | 1.38 | 7.59 |
| Jordan | 208.26 | 0.50 | 104.12 | 58.66 | 31.54 | 0.78 | 4.10 | 1.70 | 0.15 | 1.32 | 1.00 | 4.37 |
| Jamaica | 206.54 | 1.81 | 70.74 | 92.65 | 18.68 | 4.52 | 4.91 | 2.40 | 2.06 | 1.71 | 1.30 | 5.75 |
| Venezuela | 204.85 | 0.56 | 120.98 | 50.64 | 18.52 | 2.82 | 3.93 | 2.30 | 0.52 | 0.46 | 1.23 | 2.91 |
| Saint Lucia | 204.62 | 2.86 | 66.46 | 74.60 | 18.91 | 10.46 | 5.80 | 5.27 | 3.00 | 2.84 | 2.30 | 12.12 |
| Bolivia | 204.30 | 3.22 | 108.75 | 62.14 | 10.94 | 2.39 | 5.83 | 2.25 | 0.16 | 1.41 | 1.12 | 6.10 |
| Equatorial Guinea | 202.81 | 2.31 | 82.22 | 61.46 | 26.38 | 6.76 | 6.19 | 3.21 | 2.40 | 1.94 | 1.95 | 8.00 |
| Myanmar | 202.10 | 2.16 | 84.14 | 92.11 | 10.18 | 2.90 | 2.46 | 1.29 | 0.07 | 1.17 | 0.58 | 5.04 |
| Brunei | 201.28 | 2.68 | 105.19 | 57.24 | 7.74 | 8.60 | 5.68 | 5.27 | 0.82 | 1.70 | 2.14 | 4.22 |
| South Africa | 200.38 | 2.43 | 83.47 | 65.76 | 25.58 | 8.03 | 3.32 | 2.35 | 2.12 | 0.91 | 1.50 | 4.91 |
| Greenland | 199.94 | 2.19 | 90.50 | 69.65 | 4.79 | 9.87 | 8.89 | 2.19 | 1.56 | 1.49 | 4.44 | 4.37 |
| Paraguay | 199.13 | 0.89 | 99.67 | 66.97 | 13.82 | 2.06 | 4.79 | 3.12 | 0.36 | 1.29 | 2.06 | 4.11 |
| Sri Lanka | 197.09 | 1.55 | 107.93 | 58.48 | 8.41 | 7.61 | 3.49 | 1.75 | 0.16 | 1.77 | 1.08 | 4.86 |
| Northern Mariana Islands | 194.99 | 3.08 | 94.36 | 68.59 | 5.56 | 5.52 | 5.45 | 2.37 | 0.67 | 1.90 | 2.27 | 5.23 |
| Antigua and Barbuda | 191.51 | 1.88 | 75.58 | 59.35 | 22.03 | 6.18 | 5.74 | 2.02 | 2.00 | 1.62 | 2.14 | 12.97 |
| Rwanda | 191.38 | 2.07 | 61.18 | 76.04 | 31.75 | 4.20 | 3.91 | 1.62 | 0.68 | 1.21 | 1.43 | 7.29 |
| Argentina | 191.03 | 5.49 | 97.23 | 43.26 | 11.04 | 7.54 | 4.65 | 4.64 | 0.45 | 2.75 | 3.68 | 10.29 |
| Cuba | 190.97 | 1.64 | 107.16 | 50.44 | 9.98 | 5.11 | 3.75 | 3.82 | 2.78 | 0.73 | 1.76 | 3.80 |
| Ethiopia | 182.63 | 2.23 | 82.60 | 62.39 | 19.86 | 3.31 | 3.04 | 1.34 | 0.34 | 0.94 | 1.06 | 5.51 |
| Cape Verde | 182.22 | 1.85 | 101.98 | 47.69 | 6.31 | 1.63 | 4.65 | 1.29 | 0.42 | 1.29 | 0.90 | 14.19 |
| Nigeria | 181.01 | 1.96 | 79.15 | 68.21 | 8.20 | 3.52 | 4.57 | 1.53 | 0.55 | 1.55 | 0.71 | 11.06 |
| Brazil | 177.96 | 1.19 | 80.02 | 56.58 | 10.65 | 8.59 | 4.84 | 4.49 | 1.75 | 1.37 | 1.82 | 6.66 |
| Belize | 176.96 | 2.06 | 79.31 | 50.35 | 17.53 | 7.14 | 4.29 | 1.68 | 0.65 | 1.07 | 2.54 | 10.35 |
| Qatar | 176.69 | 0.58 | 122.88 | 35.29 | 2.49 | 2.27 | 6.49 | 1.24 | 0.31 | 0.49 | 1.10 | 3.54 |
| Greece | 175.69 | 0.95 | 92.48 | 54.88 | 6.08 | 3.65 | 5.00 | 4.23 | 0.34 | 1.16 | 3.28 | 3.63 |
| Turkey | 171.29 | 0.52 | 98.28 | 44.75 | 13.30 | 1.17 | 1.92 | 2.51 | 0.88 | 1.40 | 2.00 | 4.55 |
| Barbados | 170.05 | 1.91 | 62.94 | 60.01 | 11.93 | 6.82 | 4.89 | 2.38 | 5.12 | 2.27 | 1.70 | 10.08 |
| Malta | 168.71 | 1.61 | 104.33 | 35.83 | 4.63 | 4.54 | 4.92 | 2.33 | 2.31 | 1.83 | 2.66 | 3.72 |
| El Salvador | 167.29 | 0.75 | 109.59 | 34.24 | 8.86 | 1.08 | 4.47 | 1.30 | 0.10 | 0.82 | 0.43 | 5.67 |
| Maldives | 164.91 | 1.79 | 102.70 | 40.71 | 6.70 | 1.81 | 2.98 | 2.19 | 0.69 | 0.84 | 1.35 | 3.12 |
| Uruguay | 160.71 | 2.45 | 68.17 | 53.58 | 7.87 | 5.15 | 4.54 | 4.97 | 0.12 | 2.70 | 4.62 | 6.52 |
| Germany | 156.14 | 2.20 | 84.76 | 28.62 | 10.02 | 5.61 | 6.87 | 2.50 | 2.66 | 1.49 | 5.55 | 5.85 |
| Guatemala | 155.90 | 0.67 | 92.46 | 43.59 | 8.24 | 1.35 | 2.95 | 1.12 | 0.09 | 0.94 | 0.41 | 4.11 |
| Finland | 153.51 | 0.49 | 88.60 | 32.53 | 7.15 | 5.42 | 5.18 | 4.44 | 1.45 | 0.42 | 4.80 | 3.03 |
| Slovenia | 153.49 | 1.81 | 69.58 | 38.90 | 12.07 | 13.27 | 4.65 | 2.41 | 0.83 | 0.33 | 6.09 | 3.54 |
| Mexico | 152.78 | 1.05 | 95.85 | 34.47 | 8.80 | 1.39 | 4.53 | 0.96 | 0.78 | 0.63 | 0.90 | 3.42 |
| Bahrain | 151.69 | 0.95 | 99.58 | 29.27 | 4.23 | 3.09 | 6.41 | 0.95 | 0.92 | 0.80 | 1.20 | 4.29 |
| United States | 151.09 | 1.88 | 88.62 | 28.60 | 7.57 | 5.78 | 4.87 | 2.32 | 2.55 | 1.69 | 3.48 | 3.73 |
| Austria | 145.18 | 1.62 | 82.44 | 22.65 | 10.73 | 7.56 | 6.30 | 2.33 | 2.77 | 0.84 | 4.87 | 3.07 |
| Cyprus | 141.17 | 2.45 | 79.02 | 30.83 | 5.73 | 3.45 | 5.15 | 4.09 | 0.64 | 1.33 | 5.38 | 3.11 |
| Ecuador | 140.45 | 1.85 | 66.42 | 39.62 | 18.14 | 1.82 | 4.76 | 1.97 | 0.15 | 0.93 | 1.20 | 3.59 |
| Bermuda | 139.55 | 0.76 | 74.12 | 29.90 | 6.43 | 4.88 | 4.22 | 5.10 | 2.95 | 1.09 | 3.94 | 6.15 |
| Costa Rica | 137.97 | 1.14 | 77.86 | 30.36 | 11.52 | 4.21 | 3.40 | 2.62 | 0.44 | 1.24 | 1.56 | 3.62 |
| Nicaragua | 137.02 | 0.56 | 81.09 | 33.92 | 11.38 | 1.66 | 3.70 | 0.86 | 0.13 | 0.59 | 0.41 | 2.70 |
| Sweden | 133.98 | 1.08 | 72.41 | 29.53 | 4.57 | 3.32 | 8.91 | 4.50 | 0.88 | 0.99 | 4.17 | 3.62 |
| Kuwait | 132.24 | 0.41 | 80.96 | 30.00 | 11.67 | 0.96 | 1.41 | 1.30 | 0.08 | 0.82 | 0.93 | 3.69 |
| New Zealand | 128.80 | 1.96 | 70.57 | 28.41 | 2.52 | 4.01 | 6.80 | 4.73 | 2.55 | 0.59 | 5.18 | 1.48 |
| Panama | 128.35 | 0.75 | 61.74 | 42.87 | 6.53 | 4.35 | 3.64 | 1.82 | 0.99 | 1.10 | 0.74 | 3.81 |
| Luxembourg | 128.28 | 1.46 | 60.57 | 30.24 | 3.79 | 3.79 | 6.98 | 3.14 | 1.09 | 2.05 | 5.49 | 9.67 |
| Chile | 127.99 | 1.52 | 52.47 | 43.82 | 11.00 | 3.54 | 5.68 | 2.80 | 1.19 | 1.11 | 1.89 | 2.98 |
| Portugal | 127.84 | 1.45 | 48.32 | 53.85 | 4.56 | 2.86 | 3.90 | 1.79 | 2.26 | 1.53 | 3.15 | 4.18 |
| Ireland | 126.46 | 0.97 | 70.90 | 27.91 | 2.07 | 4.18 | 5.92 | 4.05 | 3.04 | 0.91 | 3.07 | 3.43 |
| Colombia | 124.24 | 0.42 | 71.20 | 28.71 | 8.88 | 1.96 | 3.35 | 2.51 | 0.64 | 1.07 | 1.83 | 3.67 |
| United Kingdom | 122.14 | 1.02 | 61.30 | 31.29 | 2.83 | 3.14 | 5.57 | 5.37 | 1.42 | 1.21 | 3.55 | 5.43 |
| Iceland | 117.99 | 0.83 | 71.10 | 23.49 | 2.98 | 1.58 | 6.69 | 3.05 | 0.55 | 0.64 | 4.12 | 2.96 |
| Belgium | 114.90 | 1.68 | 55.89 | 29.08 | 2.25 | 4.11 | 5.36 | 2.82 | 0.94 | 2.00 | 4.61 | 6.15 |
| Denmark | 114.77 | 0.95 | 54.52 | 32.25 | 1.91 | 2.33 | 6.90 | 4.91 | 1.91 | 0.96 | 4.68 | 3.44 |
| Norway | 114.32 | 0.99 | 58.33 | 28.09 | 1.61 | 2.46 | 7.19 | 5.12 | 1.35 | 1.15 | 5.35 | 2.68 |
| Italy | 113.15 | 1.59 | 51.39 | 30.42 | 10.61 | 4.09 | 4.82 | 2.35 | 1.57 | 1.08 | 2.86 | 2.37 |
| Thailand | 109.86 | 0.57 | 47.68 | 45.90 | 2.06 | 0.76 | 3.30 | 1.77 | 0.08 | 3.21 | 0.53 | 4.00 |
| Netherlands | 109.36 | 1.25 | 49.51 | 29.33 | 2.27 | 3.39 | 5.13 | 4.06 | 2.76 | 2.24 | 5.28 | 4.13 |
| Andorra | 109.13 | 1.01 | 53.84 | 27.64 | 2.16 | 3.20 | 5.71 | 4.78 | 1.42 | 1.16 | 3.47 | 4.76 |
| Puerto Rico | 108.09 | 0.98 | 57.71 | 23.39 | 10.42 | 2.70 | 3.91 | 1.12 | 1.58 | 1.22 | 1.55 | 3.51 |
| Australia | 107.79 | 1.14 | 57.48 | 25.25 | 2.35 | 3.31 | 6.54 | 2.89 | 2.53 | 1.04 | 3.02 | 2.24 |
| Canada | 105.60 | 1.35 | 63.34 | 23.03 | 1.58 | 2.18 | 3.78 | 2.62 | 1.78 | 0.96 | 2.54 | 2.43 |
| Taiwan(province of china) | 103.96 | 0.86 | 45.60 | 35.56 | 8.71 | 1.67 | 3.22 | 2.61 | 0.13 | 1.85 | 1.16 | 2.57 |
| Switzerland | 99.74 | 0.83 | 53.39 | 19.51 | 6.71 | 2.55 | 3.41 | 2.80 | 1.01 | 2.73 | 3.36 | 3.44 |
| Spain | 99.40 | 1.98 | 45.43 | 25.25 | 4.26 | 4.79 | 5.04 | 2.27 | 1.32 | 1.58 | 3.79 | 3.70 |
| Israel | 93.32 | 1.67 | 47.65 | 23.92 | 1.74 | 1.74 | 4.62 | 1.57 | 1.87 | 1.02 | 3.81 | 3.71 |
| Singapore | 92.24 | 0.39 | 51.64 | 22.03 | 9.02 | 2.27 | 1.77 | 1.83 | 0.46 | 0.94 | 0.62 | 1.27 |
| France | 86.06 | 1.48 | 38.27 | 21.34 | 2.80 | 3.44 | 4.52 | 2.08 | 0.64 | 1.69 | 3.31 | 6.49 |
| South Korea | 86.00 | 0.51 | 32.03 | 38.87 | 5.70 | 1.43 | 2.49 | 1.63 | 0.28 | 0.83 | 0.87 | 1.37 |
| Peru | 85.75 | 0.96 | 46.84 | 24.62 | 3.25 | 0.87 | 3.67 | 1.07 | 0.12 | 0.91 | 0.37 | 3.06 |
| Japan | 79.37 | 1.05 | 32.97 | 30.01 | 2.09 | 2.08 | 2.42 | 4.32 | 0.23 | 0.82 | 1.86 | 1.53 |
